# Genome-wide identification of soybean microRNAs and their targets reveals their organ-specificity and responses to phosphate starvation

**DOI:** 10.1186/1471-2164-14-66

**Published:** 2013-01-31

**Authors:** Feng Xu, Qian Liu, Luying Chen, Jiebin Kuang, Thomas Walk, Jinxiang Wang, Hong Liao

**Affiliations:** 1 State Key Laboratory for Conservation and Utilization of Subtropical Agro-bioresources, South China Agricultural University, Guangzhou 510642, PR China; 2 Root Biology Center, College of Natural Resources and Environment, South China Agricultural University, Guangzhou 510642, PR China

**Keywords:** MicroRNA, Soybean, Phosphorus, Root, Leaf, Genome, Degradome, RLM-5’ RACE, Deep sequencing

## Abstract

**Background:**

Phosphorus (P) plays important roles in plant growth and development. MicroRNAs involved in P signaling have been identified in Arabidopsis and rice, but P-responsive microRNAs and their targets in soybean leaves and roots are poorly understood.

**Results:**

Using high-throughput sequencing-by-synthesis (SBS) technology, we sequenced four small RNA libraries from leaves and roots grown under phosphate (Pi)-sufficient (+Pi) and Pi-depleted (-Pi) conditions, respectively, and one RNA degradome library from Pi-depleted roots at the genome-wide level. Each library generated ∼21.45−28.63 million short sequences, resulting in ∼20.56−27.08 million clean reads. From those sequences, a total of 126 miRNAs, with 154 gene targets were computationally predicted. This included 92 new miRNA candidates with 20-23 nucleotides that were perfectly matched to the *Glycine max* genome 1.0, 70 of which belong to 21 miRNA families and the remaining 22 miRNA unassigned into any existing miRNA family in miRBase 18.0. Under both +Pi and -Pi conditions, 112 of 126 total miRNAs (89%) were expressed in both leaves and roots. Under +Pi conditions, 12 leaf- and 2 root-specific miRNAs were detected; while under -Pi conditions, 10 leaf- and 4 root-specific miRNAs were identified. Collectively, 25 miRNAs were induced and 11 miRNAs were repressed by Pi starvation in soybean. Then, stem-loop real-time PCR confirmed expression of four selected P-responsive miRNAs, and RLM-5’ RACE confirmed that a PHO2 and GmPT5, a kelch-domain containing protein, and a Myb transcription factor, respectively are targets of miR399, miR2111, and miR159e-3p. Finally, P-responsive *cis*-elements in the promoter regions of soybean miRNA genes were analyzed at the genome-wide scale.

**Conclusions:**

Leaf- and root-specific miRNAs, and P-responsive miRNAs in soybean were identified genome-wide. A total of 154 target genes of miRNAs were predicted via degradome sequencing and computational analyses. The targets of miR399, miR2111, and miR159e-3p were confirmed. Taken together, our study implies the important roles of miRNAs in P signaling and provides clues for deciphering the functions for microRNA/target modules in soybean.

## Background

Non-coding small RNAs can be grouped into small interfering RNAs (siRNAs) and microRNAs (miRNA) in plants [1]. SiRNAs consist of trans-acting siRNA(ta-siRNA), natural antisense transcript-derived siRNA(nat-siRNA), and repeat associated siRNA (ra-siRNA) [1]. Increasing evidences verify that 20–24 nucleotide-long miRNAs play important roles in growth, development, and stress adaptations in planta via modulating gene activity [1,2]. In the plant cell, primary miRNA (pri-miRNA) is transcribed by RNA polymerase II (PolII) [3], then cut to precursor of miRNA (pre-miRNA) containing the distinctive stem-loop structure by Dicer-like (DCL), and finally pre-miRNA is processed to form miRNA/miRNA* duplexes, with miRNA* ultimately degraded [1,4]. Hua Enhancer 1 (HEN1) methylates the miRNA/miRNA* duplex on the 3’terminal nucleotide of each strand to protect it from degradation by other small exonucleases. The methylated duplex is transported into the cytoplasm with the help of HASTY (HST) [1] and recruited by ARGONAUTE (AGO) proteins to suppress translation or to cleave the target transcripts mostly in the coding region [1].

Like DCLs, AGOs are highly conserved proteins across plants and animals. In Arabidopsis, ten genes encode AGO, which are grouped into 4 clades [1,5]. AGO1 is involved in most miRNA biogenesis. AGO4 and AGO6 are responsible for ra-siRNA production and control DNA methylation. AGO7 participates in ta-siRNA formation [5]. Recent studies showed that the 5’ terminal nucleotide appears to determine the fate of miRNAs. For instance, AGO1 preferentially binds miRNAs with a 5’ terminal uridine (U), and AGO2 and AGO4 recruit small RNAs with 5’ terminal adenosine (A), AGO5 binds small RNAs that terminate with cytosine (C) at the 5’ terminus [5].

Recently, a number of miRNAs have been documented to be involved in nutrient signaling. Phosphorus (P) deficiency specifically induces miR399 in Arabidopsis and rice [6-8]. MiR827 induced by P limitation negatively regulates the transcript level of NITROGEN LIMITATION ADAPTATION (NLA) in Arabidopsis. Plus, osa-miR827 expression is strongly enhanced by phosphate (Pi) starvation in both roots and leaves. *In situ* hybridization indicates that osa-miR827 is expressed in mesophyll, epidermis and ground tissues of roots. Moreover osa-miR827 relays P signaling via negatively regulating the target genes of OsSPX-MFS1 and OsSPX-MFS2 [9]. More ambiguous results pertaining to miRNA involvement in P deprivation was obtained with the observation through RNA ligase mediated 5’rapid amplification of cDNA ends (RLM-5’ RACE) that miR2111 cleaves *At3g27150*, which encodes a kelch domain-containing F-box protein, but Pi starvation induced the levels of both miR2111 and *At3g27150*[10]. One last set of results in regards to P nutrition shows that miR778, miR398, miR169 and miR408 are also responsive to P limitation [10,11]. In regards to other nutrients, miR167 and miR393 were found to regulate root development in response to nitrogen (N) [12,13]. Sulphur (S) starvation stimulated miR395, which targets plastidic ATP sulfurylase (APS), and the high-affinity sulfate transporter SULTR2;1 [6,12,14]. Copper (Cu) limitation stimulates the level of miR398 and miR402 [6,15,16]. Once expressed, miRNAs can be transported in phloem but not xylem vessels [12]. The levels of miR395, miR398 and miR399 were strongly augmented in response to S, Cu or Pi starvation in phloem. Under iron (Fe) deficiency, while the levels of miR399 and miR2111 were decreased in phloem, and even undetectable in roots and leaves [12,16], indicating that Fe limitation alters P homeostasis.

The first study on soybean miRNAs in 2008 identified 35 novel miRNA families [17], and then sixty-nine miRNAs grouped into 33 families and their targets were determined through computational analysis [18]. After that, eighty-seven novel miRNAs were further isolated from soybean roots, seeds, flowers and nodules [19]. Twenty-six new miRNAs were identified via small RNA and degradome-associated deep sequencing in soybean seeds [20]. In addition, mis-expressions of miR482, miR1512, and miR1515 in soybean increased nodulation [21].

Low P availability resulting from its low mobility in soils is a common limiting factor for soybean yield [22]. Although miR399, the classic P-responsive miRNA, was predicted to exist in soybean [18], no experiments to verify the existence of this or other P-responsive mRNAs have been reported in soybean. To identify P-responsive miRNAs, along with, leaf- and root-specific miRNAs in soybean, in this study we sequenced four small RNA libraries from soybean leaves and roots grown under Pi-sufficient (+Pi) and Pi-depleted (-Pi) conditions, respectively. From those libraries, new conserved, less-conserved, and novel miRNAs as well as P-responsive miRNAs in soybean leaves and roots were identified genome-wide. Furthermore the targets of soybean miRNAs were determined via degradome sequencing from -Pi-roots combined with computational analyses. Finally, the existence of four P-responsive miRNAs (miR399, nov_6, nov_9, and nov_10) were verified via stem-loop real time (RT) PCR, and the targets of miR399, miR2111, and miR159e-3p were confirmed via RLM 5’ RACE. With results in hand, the possible functions of miRNA/target modules are discussed.

## Results

### Deep sequencing of small RNAs in soybean leaves and roots

Relative to control plants, long-term (7 days or 14 days) Pi starvation increased the ratio of roots to shoots (Additional file 1A), and decreased the concentration of soluble phosphate (SPi) in leaves and roots (Additional file 1B). Pi limitation globally induces the expression of many genes, including *AtIPS1* (*At3g09922*) a well known non-coding Pi-starvation responsive gene, and *AtPLDZ2* (*At3g05630*), which is involved in the conversion of phospholipid to glycolipid and free P [7]. Based on Blast searches of these genes in Phytozome ( http://www.phytozome.org) and TIGR ( http://www.tigr.org), the sequences of *GmIPS1* and *GmPLDZ2* were retrieved. The transcript levels of *GmPLDZ2* (Additional file 1C) and *GmIPS1* (Additional file 1D) were induced by long-term Pi deprivation. These data verified that the Pi starvation in soybean was affected at both physiological and molecular levels.

Additional file 1 indicated that soybean was subjected to Pi starvation at 6 h, 12 h, 7 d and 14 d treatments. Accordingly, the 6 h and 12 h Pi starvation were treated as short-term P limitation, and 7 d and 14 d as long-term P stress. To augment the chance of finding more miRNAs in a single sequencing run, total RNA was extracted from leaves and roots of both +Pi and -Pi plants at 6 h, 12 h, 7 d, and 14 d, and then equal amounts of total RNA from each time point and treatment were pooled and used to construct four small RNA libraries labeled as leaf+Pi (HPL), root+Pi (HPR), leaf-Pi (LPL) and root-Pi (LPR).

Overall, more than 20 million raw reads from the four small RNA libraries were obtained. Clean reads were produced by excluding reads smaller than 18 nt and adaptors. The percentage of clean reads to total reads ranged from 96.92% to 99.59% (Table 1). More than 80% and 75% of total RNA reads from the two leaf libraries and two root libraries were mapped to soybean genome, respectively (Table 1). Here, unique small RNAs were defined as small RNA species with unique sequence. For unique small RNAs, more than 80% of reads in HPL and LPL libraries, and more than 55% of reads in HPR and LPR libraries could be mapped to the genome, respectively (Table 1).

**Table 1:** Statistics of four small RNA libraries from soybean

	**Library name**^**a**^			
	**Leaf+Pi (HPL)**	**Leaf-Pi (LPL)**	**Root+Pi (HPR)**	**Root-Pi (LPR)**
Total reads	22925183	26658394	21456069	28635677
High quality reads	22643245(100%)	26377550(100%)	20957370(100%)	27944092(100%)
Reads smaller than 18nt	126772(0.56%)	68893(0.26%)	347577(1.66%)	800247(2.86%)
Clean reads	22483373(99.29%)	26268210(99.59%)	20564547(98.13%)	27083979(96.92%)
Total small RNA reads mapping to genome^b^	18248364/22483373 (81.16%)	22176679/26268210 (84.42%)	20591098/27083979 (76.03%)	15573680/20564547 (75.73%)
Unique small RNA reads mapping to genome^b^	2396442/2901995 (82.58%)	3301579/3951946 (83.54%)	2360847/4071174 (57.99%)	2583592/4641188 (55.67%)

^a^Four small RNA libraries were constructed. Total RNA were extracted from soybean leaves and roots grown in +Pi or -Pi nutrient solutions.

Accordingly the small RNA libraries were named leaf+Pi (HPL), leaf-Pi (LPL), root+Pi (HPR), and root-Pi (LPR).

^b^Total small RNA reads and unique small RNA reads were mapped to the *Glycine max* genome v1.0 sequences available in Phytozome ( http://www.phytozome.org).

Additional file 2 summarized the origin profiles of total small RNAs. Fifteen and 14 percent of clean small RNA reads were mapped to known miRNA genes in HPR and LPR, respectively, but over 48% of small RNA reads from HPL and LPL libraries were mapped to miRNA genes. Additional file 3 demonstrated that the percentage of clean small RNAs with 20-24 nt was 85.16%, 89.90%, 52.22%, and 46.05% in HPL, LPL, HPR, and LPR, respectively, indicating good quality for the four small RNA libraries. In addition, more than 20% of the clean small RNA reads were assigned to unannotated regions of the *Glycine max* 1.0 genome, supporting the notion that most small RNAs originate from intergenic regions.

**Figure 1:**
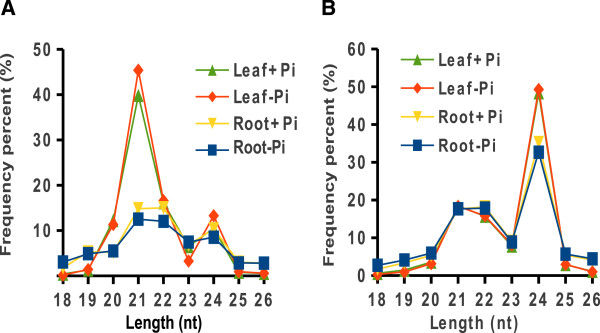
**Size distribution of small RNAs through Solexa sequencing.** Small RNA size distribution from the libraries of leaves and roots grown under +Pi (HP) and -Pi (LP) conditions. The size distribution was plotted versus frequency (%) of small RNA read length relative to total small RNA reads (**A**) or unique small RNA reads (**B**).

Small RNAs containing 21, 22, or 24 nucleotides had high abundance relative to those of other lengths in all four libraries, while those containing 20 nucleotides were high in leaves (Figure 1A; Additional file 3). The 39-45% abundance of 21 nt small RNAs in HPL and LPL library was dominant over 22 and 24-nt small RNAs (Figure 1A). When only unique reads were considered, 24-nt small RNAs were most abundant, with 21 nt and 22 nt small RNA following in all libraries (Figure 1B). Overall, these data are consistent with previous studies [10,20].

### The nucleotide preference of small RNAs

As described above (see Background), AGO proteins preferentially recruit miRNAs based upon the 5’ terminal nucleotide [5]. In the sequences obtained from soybean, the representation of the nucleotides in the 5’ position varied among miRNA lengths (Additional file 4).

Over 90% of the first nucleotide in 18 nt small RNAs in the two root libraries was C. The percentage of guanosine (G) in the first base of 19 nt small RNAs was the highest in the LPR library, whereas that of A was the highest in the HPR library. Interestingly, greater than 50% of U in the first position of 20 nt, 21 nt, and 22 nt-long small RNAs in all four libraries. Over 60% and 90% of 23 nt small RNAs were 5’-terminated with U in the two root libraries, but more than 90% of 23 nt small RNAs were tagged with G in the two leaf libraries. The percentage of A and U in the first position of 24 nt small RNAs were higher than that of the other nucleotides in leaf libraries, and the percentage of G in the first position of 24 nt small RNAs was higher than other nucleotides in the two root small RNA libraries. The 25 nt small RNAs with 5’ U were dominant in HPL, LPL, and HPR libraries (Additional file 4). These results indicate that P availability generally played little roles in the first base bias of small RNAs in soybean.

### Identification of conserved and new miRNAs

After excluding the small RNA reads which can be mapped to protein-coding and structural RNA-coding regions, candidate miRNAs were predicted based on published methods [23]. To determine *bona fide* miRNAs, several strict criteria to scrutinize mature miRNAs were implemented, including: (1) the abundance of putative miRNAs was at least 100 transcripts per million (TPM) in any one of the four libraries; (2) the reads for 5p and 3p strand could be detected and the ratio of 5p/3p or 3p/5p was higher than 0.9; (3) the length of small RNAs was 20 to 24 nt; (4) the minimum folding free energy of the precursor of miRNA was lower than -37 KJ/mol. (5) the minimal free energy index (MFEIs) was higher than 0.85; (6) RNAfold predicted a hairpin secondary structure for pre-miRNA; (7) features of real pre-miRNA, as tested in the online service, plantMIRNAPred [24], verified the candidate as pre-miRNA. Subsequently, candidate mature miRNAs were Blast searched against the soybean miRNAs deposited in miRBase 18.0 ( http://www.mirbase.org) and new conserved, less-conserved (fabaceae-specific) and soybean novel microRNAs (soybean-specific) were determined.

As shown in Table 2, seventeen soybean miRNA families have been found in other plant families, and six have only been noted in fabaceae. Mature miRNAs ranged from 20 to 23 nt, and 11 out of 23 miRNA families were 21 nt-long. Most of the first nucleotide in all soybean miRNAs were U. More C was present in the 19th nucleotide than any other nucleotide (Table 2). This was consistent with earlier studies on soybean and cotton [18]. At the time of conducting this research, more than 240 soybean miRNAs, representing 65 families were curated in miRBase. A total of 126 soybean miRNAs in the current work were identified, 34 of which were annotated in miRBase 18.0. Eighty-nine of the 126 miRNAs, grouped into 21 families were conserved (Table 3), and 25 were less conserved miRNAs that were only be found in legumes, namely legume-specific (Table 4). The remaining 12 were soybean-specific (novel) miRNAs (Table 5).

**Table 2:** Conserved and less-conserved miRNAs families detected in four small RNA libraries from soybean

**Family**	**Size (nt)**	**Site (5’ to 3’)**	**Homology in plant species**					
		**1st**	**19th**	***A.thaliana***	***O.sativa***	***Z.mays***	***M.truncatula***	***P.trichocarpa***
**Conserved**^**a**^								
MIR156	20-21, 23	U (82%)	A (59%)	+	+	+	+	+
MIR159	21	U (100%)	C (100%)	+	+	+	+	+
MIR160	21	G (100%)	A (100%)	+	+	+	+	+
MIR162_1	21	U (100%)	C (100%)	+	−	−	+	+
MIR164	21	U (100%)	G (100%)	+	+	+	+	+
MIR166	20-22	U (80%)	C (50%)	+	+	+	+	+
MIR167_1	21-22	U (100%)	C (100%)	+	+	+	+	+
MIR168	20-21	C (67%)	A (67%)	+	+	+	+	+
MIR169_2	21	A (67%)	C (100%)	+	+	+	+	+
MIR172	21	U/A/G (33%)	C (67%)	+	+	+	+	+
MIR390	21	A (100%)	G (75%)	+	+	+	+	+
MIR396	21	G (60%)	A (60%)	+	+	+	+	+
MIR397	21	U (100%)	A (100%)	+	+	+	−	+
MIR399	21	U (100%)	C (100%)	+	+	−	+	+
MIR2118	22	U (100%)	C (100%)	−	+	+	+	+
MIR408	20-21	C (67%)	A (50%)	+	+	+	+	+
MIR482	21-22	U (56%)	A (33%)	−	−	−	−	+
**Less-conserved**^**b**^								
MIR1507	22	U (100%)	C (100%)	−	−	−	+	−
MIR1508	21-22	U/C (50%)	U/G (50%)	−	−	−	−	−
MIR1509	21-22	U (100%)	G (100%)	−	−	−	+	−
MIR1510	21-22	A (75%)	U (50%)	−	−	−	+	−
MIR2109	21	U (100%)	U (100%)	−	−	−	−	−
MIR3522	22	U (100%)	U (100%)	−	−	−	−	−

The miRNAs in four soybean small RNA libraries were classified into conserved and less-conserved miRNAs based on the data in miRBase ( http://www.mirbase.org).

^a^For conserved miRNAs, the counterpart of which can be found in other reference plants apart from legume.

^b^For less-conserved miRNAs, the homology of which can only be found in legume.

^+^indicates the existence of miRNA in species, and - shows no homology in plant species.

A, adenosine; C, cytosine; G, guanosine; nt, nucleotide; U, uridine.

*A.thaliana*, *Arabidopsis thaliana*; *O.sativa*, *Oryza sativa*; *Z.mays*, *Zea mays*; *M.truncatula*, *Medicao truncatula*; *P.P.trichocarpa*, *Poplus trichocarpa*.

**Table 3:** Conserved soybean miRNAs in four small RNA libraries

**miRNA**	**Sequence (5’ to 3’)**	**Size (nt)**	**Ch**	**Start:end (+/-)**	**Arm**	**TPM in HPL**	**TPM in LPL**	**TPM in HPR**	**TPM in LPR**	**Registered in**
										**miRBase or not**
miR156d	TTGACAGAAGATAGAGAGCAC	21	8	3891352:3891504:+	5	34857	30879	18368	15715	Yes
miR156h	TGACAGAAGAGAGTGAGCAC	20	4	4990842:4990964: −	5	66744	55303	22303	26339	Yes
miR156o	TTGACAGAAGAGAGTGAGCAC	21	17	37759430:37759553:+	5	1749	1579	1271	998	Yes
miR156p	ACAGAAGATAGAGAGCACAG	20	7	9347129:9347272:+	5	46	47	94	131	No
miR156q	TGACAGAAGATAGAGAGCAC	20	19	8895390:8895494:+	5	483	526	403	452	No
miR156r	TGACAGAAGAGAGTGAGCACT	21	13	20521462:20521566:+	5	75	57	159	206	No
miR156s	TGACAGAAGAGAGTGAGCACT	21	17	4291649:4291772: −	5	75	57	159	206	No
miR156t	TGACAGAAGAGAGTGAGCACA	21	2	41864154:41864278:+	5	173	156	99	108	No
miR156u	TGACAGAAGAGAGTGAGCACA	21	4	4257047:4257175:+	5	173	156	99	108	No
miR156v	TGACAGAAGAGAGTGAGCACA	21	6	4013560:4013688:+	5	173	156	99	108	No
miR156w	TGACAGAAGAGAGTGAGCACA	21	14	9431588:9431718:+	5	173	156	99	108	No
miR156x	TGACAGAAGAGAGTGAGCACA	21	17	38431855:38431985: −	5	173	156	99	108	No
miR156y	CTGACAGAAGATAGAGAGCAC	21	18	61442592:61442691: −	5	28	27	196	106	No
miR156z	GCTCACTACTCTTTCTGTCGGTT	23	19	40699080:40699213: −	3	503	491	12	17	No
miR156_c1	TTGACAGAAGAAAGGGAGCAC	21	1	55282671:55282770:+	5	291	274	0.01	0.01	No
miR156_c2	TTGACAGAAGAAAGGGAGCAC	21	11	453213:453312: −	5	291	274	0.01	0.01	No
miR156_c3	TTGACAGAAGAGAGAGAGCAC	21	2	50779230:50779334: −	5	182	139	45	32	No
miR159e-3p	TTTGGATTGAAGGGAGCTCTA	21	7	9524916:9525128: −	5	191	250	293	109	Yes
miR160f	GCGTATGAGGAGCCAAGCATA	21	10	43851636:43851757: −	3	56	46	117	128	No
miR160g	GCGTATGAGGAGCCAAGCATA	21	20	40554887:40554987:+	3	56	46	117	128	No
miR162c	TCGATAAACCTCTGCATCCAG	21	17	10181487:10181612:+	3	97	111	59	54	Yes
miR164e	TGGAGAAGCAGGGCACGTGCA	21	2	1511590:1511686:+	5	1621	2713	969	1532	No
miR164f	TGGAGAAGCAGGGCACGTGCA	21	3	45537767:45537877:+	5	1621	2713	969	1532	No
miR164g	TGGAGAAGCAGGGCACGTGCA	21	3	46896220:46896314:+	5	1621	2713	969	1532	No
miR164h	TGGAGAAGCAGGGCACGTGCA	21	19	48157202:48157297:+	5	1621	2713	969	1532	No
miR164i	TGGAGAAGCAGGGCACGTGCA	21	20	45788090:45788206: −	5	1621	2713	969	1532	No
miR166a-5p	GGAATGTTGTCTGGCTCGAGG	21	16	1912569:1912713: −	5	171	362	89	110	Yes
miR166g	TCGGACCAGGCTTCATTCCCC	21	10	2905308:2905432: −	3	16705	19874	20693	18766	Yes
miR166h-3p	TCTCGGACCAGGCTTCATTCC	21	8	14990535:14990743:+	3	520	620	5834	5908	Yes
miR166j-3p	TCGGACCAGGCTTCATTCCCG	21	15	3688752:3688943: −	3	964	1127	3563	3670	Yes
miR166k	TCGGACCAGGCTTCATTCCCT	21	6	10985730:10985884:+	3	1128	1461	2223	2182	No
miR166l	TCTCGGACCAGGCTTCATTC	20	16	3661371:3661614:+	3	48	32	649	549	No
miR166m	TCTCGGACCAGGCTTCATTC	20	19	36649690:36649989: −	3	48	32	649	549	No
miR166n	TTTCGGACCAGGCTTCATTCC	21	3	39519830:39519954: −	3	72	68	148	113	No
miR166o	TCGGACCAGGCTTCATTCCC	20	6	12992803:12992972: −	3	75	53	118	103	No
miR166p	TCGGACCAGGCTTCATTCCC	20	7	10198823:10198944:+	3	75	53	118	103	No
miR166q	TCGGACCAGGCTTCATTCCC	20	9	37125221:37125362: −	3	75	53	118	103	No
miR166r	GGAATGTCGTCTGGTTCGAGA	21	2	14340754:14340878:+	5	120	116	12	15	No
miR166s	TTCGGACCAGGCTTCATTCCCC	22	5	37747448:37747571: −	3	163	192	106	100	No
miR166t	TTCGGACCAGGCTTCATTCCCC	22	8	282637:282760: −	3	163	192	106	100	No
miR166u	GGAATGTTGTCTGGCTCGAGG	21	6	12992922:12993135: −	5	177	370	92	114	No
miR167e	TGAAGCTGCCAGCATGATCTT	21	20	37901894:37902003:+	5	1866	1676	2108	1984	Yes
miR167g	TGAAGCTGCCAGCATGATCTGA	22	10	39044864:39044969:+	5	160	139	12	11	Yes
miR167j	TGAAGCTGCCAGCATGATCTG	21	20	44765083:44765188:+	5	5198	6009	381	405	Yes
miR167k	TGAAGCTGCCAGCATGATCTA	21	3	39319064:39319173:+	5	236	255	166	61	No
miR167l	TGAAGCTGCCAGCATGATCTTA	22	10	46574251:46574360: −	5	1210	1096	951	839	No
miR168	TCGCTTGGTGCAGGTCGGGAA	21	9	41353223:41353352: −	5	10469	8526	7573	7864	Yes
miR168c	CCCGCCTTGCATCAACTGAAT	21	1	48070300:48070429: −	3	195	223	277	327	No
miR168d	CCCGCCTTGCATCAACTGAAT	21	9	41353223:41353352: −	3	195	223	277	327	No
miR169c	AAGCCAAGGATGACTTGCCGA	21	9	5295079:5295216:+	5	29	12	119	95	Yes
miR169f	CAGCCAAGGATGACTTGCCGG	21	10	40332781:40332933: −	5	103	99	29	32	Yes
miR169o	CAGCCAAGGGTGATTTGCCGG	21	15	14150055:14150198:+	5	72	144	301	326	No
miR169p	AAGCCAAGGATGACTTGCCGG	21	9	5299595:5299718:+	5	21	14	118	92	No
miR169q	AAGCCAAGGATGACTTGCCGA	21	15	14202454:14202568:+	5	0.01	12	125	102	No
miR169r	AAGCCAAGGATGACTTGCCGG	21	17	4864165:4864284: −	5	0.01	0.01	122	0.01	No
miR172b-3p	AGAATCTTGATGATGCTGCAT	21	13	40401672:40401822: −	3	3576	4906	584	689	Yes
miR172h-5p	GCAGCAGCATCAAGATTCACA	21	10	43474719:43474839:+	5	103	97	9	11	Yes
miR172k	TGAATCTTGATGATGCTGCAT	21	12	33560621:33560754:+	3	89	133	0.01	0.01	No
miR390b	AAGCTCAGGAGGGATAGCACC	21	2	44954748:44954865:+	5	32	28	175	199	Yes
miR390d	AAGCTCAGGAGGGATAGCGCC	21	11	30272752:30272868:+	5	515	328	230	268	No
miR390e	AAGCTCAGGAGGGATAGCGCC	21	18	53278026:53278171:+	5	515	328	230	268	No
miR390f	AAGCTCAGGAGGGATAGCGCC	21	18	5047758:5047875: −	5	515	328	230	268	No
miR396a-5p	TTCCACAGCTTTCTTGAACTG	21	13	26338131:26338273: −	5	75	110	323	299	Yes
miR396b-3p	GCTCAAGAAAGCTGTGGGAGA	21	13	26329939:26330049:+	3	138	220	0.01	0.01	Yes
miR396c	TTCCACAGCTTTCTTGAACTT	21	13	43804787:43804882:+	5	141	191	93	101	Yes
miR396j	GTTCAATAAAGCTGTGGGAAG	21	13	26338141:26338266: −	5	0.01	130	0.01	0.01	No
miR396k	GCTCAAGAAAGCTGTGGGAGA	21	13	26329931:26330059:+	3	91	172	8	21	No
miR397a	TCATTGAGTGCAGCGTTGATG	21	8	4639045:4639154: −	5	161	253	0.01	17	Yes
miR399a	TGCCAAAGGAGATTTGCCCAG	21	5	34958613:34958732: −	3	21	712	8	280	No
miR399b	TGCCAAAGGAGATTTGCCCAG	21	5	34967642:34967778: −	3	21	712	8	280	No
miR399c	TGCCAAAGGAGATTTGCCCAG	21	8	9118500:9118624: −	3	21	712	8	280	No
miR399d	TGCCAAAGGAGATTTGCCCAG	21	8	9126508:9126640: −	3	21	712	8	280	No
miR399e	TGCCAAAGAAGATTTGCCCAG	21	5	34963165:34963300: −	3	0.01	394	0.01	0.01	No
miR2118a	TTGCCGATTCCACCCATTCCTA	22	20	35349746:35349875:+	3	2084	3812	2429	2557	Yes
miR2118b	TTGCCGATTCCACCCATTCCTA	22	10	48574023:48574132: −	3	2084	3812	2429	2557	Yes
miR408b −5p	CTGGGAACAGGCAGGGCACG	20	3	44626682:44626839: −	5	228	362	13	12	Yes
miR408c	ATGCACTGCCTCTTCCCTGGC	21	10	36556991:36557143: −	3	516	1215	70	84	Yes
miR408d	CTGGGAACAGGCAGGGCACGA	21	3	44626682:44626839: −	5	409	588	81	78	No
miR408e	CAGGGGAACAGGCAGAGCATG	21	2	837410:837560:+	5	380	295	12	14	No
miR408f	CAGGGGAACAGGCAGAGCATG	21	10	36556991:36557143: −	5	380	295	12	14	No
miR408g	GCTGGGAACAGGCAGGGCACG	21	3	44626682:44626839: −	5	100	167	21	22	No
miR482b-3p	TCTTCCCTACACCTCCCATACC	22	20	35360307:35360413:+	3	37	154	218	494	Yes
miR482e	GGAATGGGCTGATTGGGAAGC	21	2	7783811:7783923:+	5	560	711	651	562	No
miR482f	TTCCCAATTCCGCCCATTCCTA	22	2	7783811:7783923:+	3	182	696	91	163	No
miR482g	TTCCCAATTCCGCCCATTCCTA	22	18	61452897:61453007: −	3	182	696	91	163	No
miR482h	TATGGGGGGATTGGGAAGGAA	21	10	48569622:48569728: −	5	147	110	133	114	No
miR482i	TATGGGGGGATTGGGAAGGAA	21	20	35360307:35360413:+	5	147	110	133	114	No
miR482j	TTCCCAATTCCGCCCATTCCTA	22	2	7783818:7783913:+	5	195	736	0.01	186	No
miR482k	TTCCCAATTCCGCCCATTCCTA	22	18	61452907:61453000: −	5	195	736	0.01	186	No

Soybean conserved miRNAs were grouped into different miRNA families based on Blast analysis of mature miRNA sequences in miRBase ( http://www.mirbase.org). For conserved miRNAs, homology of which can be found in other plant species apart from legume.

Ch, chromosome; HPL, leaf+Pi; HPR, root+Pi; LPL, leaf-Pi; LPR, root-Pi; nt, nucleotide; TPM, transcript per million reads; +, sense strand; −, antisense strand.

miR156_c1 means new soybean miR156 candidate 1, and so on in other miRNA families.

**Table 4:** Less-conserved soybean miRNAs in four small RNA libraries

**miRNA**	**Sequence (5’ to 3’)**	**Size (nt)**	**Ch**	**Start:End (+/-)**	**Arm**	**TPM in HPL**	**TPM in LPL**	**TPM in HPR**	**TPM in LPR**	**Registered in**
										**miRBase or not**
**MIR1507**										
miR1507a	TCTCATTCCATACATCGTCTGA	22	13	25849776:25849883:+	3	93418	6787	2 73567	59118	Yes
**MIR1508**										
miR1508d	TAGAAAGGGAAATAGCAGTTG	21	9	28530172:28530267:+	3	6600	7129	1596	1456	No
miR1508e	CTAGAAAGGGAAATAGCAGTTG	22	16	32903737:32903831:+	3	1347	1516	374	317	No
**MIR1509**										
miR1509a	TTAATCAAGGAAATCACGGTCG	22	17	10099759:10099871:+	5	7221	8003	21070	17929	Yes
miR1509c	TTAATCAAGGAAATCACGGTTG	22	5	7774098:7774206: −	5	316	531	2125	2125	No
miR1509d	TTAATCAAGGAAATCACGGTC	21	17	10099759:10099871:+	5	45	50	130	117	No
**MIR1510**										
miR1510b-3p	TGTTGTTTTACCTATTCCACC	21	2	6599300:6599391:+	3	75	125	139	162	Yes
miR1510b-5p	AGGGATAGGTAAAACAACTACT	22	2	6599292:6599401:+	5	1462	3478	968	1980	Yes
miR1510c	AGGGATAGGTAAAACAACTAC	21	2	6599292:6599401:+	5	750	851	697	1377	No
miR1510d	AGGGATAGGTAAAACAATGAC	21	16	31518900:31519009:+	5	136	266	245	822	No
**MIR2109**										
miR2109a	TGCGAGTGTCTTCGCCTCTGA	21	4	28532444:28532532: −	5	50	6	181	220	No
**MIR3522**										
miR3522a	TGAGACCAAATGAGCAGCTGAC	22	15	4318787:4318887:+	5	139	115	7	0.01	No
**Family undefined**										
miR1511	AACCAGGCTCTGATACCATGG	21	18	21161229:21161335:+	3	1099	1034	5415	8419	Yes
miR1511a	AACCAGGCTCTGATACCATGGT	22	18	21161229:21161335:+	3	31	28	77	113	No
miR1512b	TAACTGGAAATTCTTAAAGCAT	22	2	8618690:8618783: −	5	0.01	8	72	102	No
miR3508	TAGAAGCTCCCCATGTTCTCA	21	15	5418778:5418967:+	3	75	355	120	104	No
miR4345	TAAGACGGAACTTACAAAGATT	22	14	49067429:49067781:+	5	77	92	151	139	Yes
miR4345a	TTAAGACGGAACTTACAAAGATT	23	14	49067429:49067781:+	5	131	158	314	260	No
miR4345b	CTAAGACGGAACTTACAAAGAT	22	14	49069094:49069198:+	5	127	141	0.01	0.01	No
miR4376-5p	TACGCAGGAGAGATGACGCTGT	22	13	40845924:40846035:+	5	250	154	0.01	0.01	Yes
miR4376a	ACGCAGGAGAGATGACGCTGT	21	13	40845924:40846035:+	5	352	155	0.01	0.01	No
miR4376b	TACGCAGGAGAGATGACGCTG	21	13	40845924:40846035:+	5	358	216	0.01	0.01	No
miR4413c	TAAGAGAATTGTAAGTCACTG	21	19	1788521:1788616: −	5	46	65	120	119	No
miR4416a	ACGGGTCGCTCTCACCTGGAG	21	2	30498955:30499126: −	3	0.01	11	123	158	No
miR4416b	ATACGGGTCGCTCTCACCTAGG	22	19	40699080:40699213: −	3	8	16	95	139	No

Soybean less-conserved miRNAs were grouped into miRNA families based on Blast analysis of mature miRNA sequences in miRBase 18.0 ( http://www.mirbase.org). For less-conserved miRNAs, homology of which can only be found in legume (legume-specific).

Ch, chromosome; HPL, leaf+Pi; HPR, root+Pi; LPL, leaf-Pi; LPR, root-Pi; nt, nucleotide; TPM, transcript per million reads; +, sense strand; −, antisense strand.

**Table 5:** Novel soybean miRNAs in four small RNA libraries

**miRNA**	**Sequence (5’ to 3’)**	**Size (nt)**	**Ch**	**Start:End (+/ −)**	**Arm**	**TPM in HPL**	**TPM in LPL**	**TPM in HPR**	**TPM in LPR**	**Registered in**
										**miRBase or not**
miRnov_1a	AAAGCCATGACTTACACACGC	21	17	1401437:1401518: −	5	160	173	271	249	No
miRnov_1b	AAAGCCATGACTTACACACGC	21	20	223678:223767: −	5	163	179	281	259	NO
miRnov_2	ATTGGGACAATACTTTAGATA	21	18	52797184:52797492:+	3	0.01	0.01	0.01	153	NO
miRnov_3	GGAGATGGGAGGGTCGGTAAAG	21	20	35349749:35349874:+	5	419	371	351	595	NO
miRnov_4	ATATGGACGAAGAGATAGGTAA	21	20	40357028:40357130:+	5	115	120	249	187	NO
miRnov_5a	CAGGGGAACAGGCAGAGCATG	21	2	837420:837549:+	5	394	307	86	15	NO
miRnov_5b	CAGGGGAACAGGCAGAGCATG	21	10	36557001:36557133: −	5	394	307	86	15	NO
miRnov_6	AGAGGTGTATGGAGTGAGAGA	21	13	25849778:25849881:+	5	256	122	96	98	NO
miRnov_7	AGCTGCTCATCTGTTCTCAGG	21	15	4318784:4318876:+	3	48	137	0.01	0.01	NO
miRnov_8	GCTCACTACTCTTTCTGTCGGTT	21	17	37759439:37759543:+	3	609	615	18	24	NO
miRnov_9	TCAATCCTGGAAGAACCGGCG	21	13	35514890:35515064:+	3	44	35	106	51	NO
miRnov_10	AGGAAGCTAAGACGGAACTTA	21	14	49069088:49069204:+	5	44	0.01	110	87	NO

Soybean novel miRNAs were identified based on Blast analysis of mature miRNA sequences. For novel miRNAs, which were not previously in miRBase and not assigned into any family were denoted by miRnov (soybean-specific).

Ch, chromosome; HPL, leaf+Pi; HPR, root+Pi; LPL, leaf-Pi; LPR, root-Pi; nt, nucleotide; TPM, transcript per million reads; +, sense strand; −, antisense strand.

Importantly, ninety-two soybean miRNAs have been identified in this study for the first time (Tables 3, 4 and 5). Sixty-two of these are conserved miRNAs and grouped into 14 families (Table 3). Eight are less-conserved and classified into 5 families (Table 4). Ten of the miRNAs identified in this study can be found in other plant species, but have not yet been classified into any miRNA families in miRBase. Finally, 12 miRNAs identified herein are as yet soybean-specific novel miRNAs (Table 5). More importantly, in regards to plant nutrition, miR399a, miR399b, miR399c, miR399d, and miR399e were detected in the present small libraries. Moreover miR399a, miR399b, and miR399e were localized to chromosome 5, while miR399c and miR399d were localized to chromosome 8 (Table 3).

### Soybean leaf- and root-specific miRNAs

**Figure 2:**
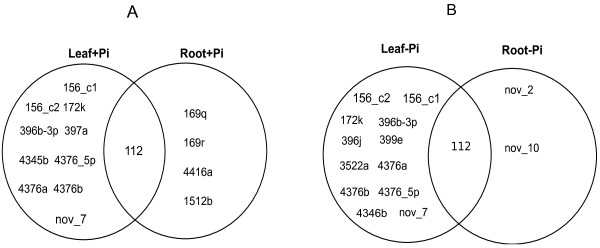
**Unique and overlapping soybean miRNAs in leaves and roots under +Pi and -Pi conditions.A**: leaf-specific and root-specific miRNAs in +Pi conditions; **B**:leaf-specific and root-specific miRNAs under -Pi conditions.

To determine leaf- and root-specific miRNAs, the abundance of each mature miRNA was compared between leaves and roots. If a miRNA was not detectable in leaves or roots, then it was considered as specific to the other tissue. Under control (+Pi) conditions, ten leaf-specific, and four root-specific miRNAs were found (Figure 2A). Under Pi-depleted conditions, there are twelve leaf-specific miRNAs, and two root-specific miRNAs (Figure 2B). The two root-specific miRNAs in Pi-depleted plants are novel to soybean (gma-nov_2 and gma-nov_10).

A number of miRNAs are notable for their expression patterns. This included most members of miR156, 164 and 167 families, along with 12 individual miRNAs (miR168, miR172b-3p, miR2118a, miR2118b, miR408c, miR1507a, miR1508d, miR1508e, miR1509a, miR1510b-5p, miR1510c, and miR1511) that were found in high abundance (>1000 TPM) in one or both of the HPL or LPL treatments (Tables 3, 4 and 5). Others appeared to be constitutively expressed in leaves and roots, including, for example, members of the conserved miR160, miR164, and miR2118 families (Table 3). Within families, expression can vary significantly among family members. For example, expression levels of miR156 family members ranged from 46 to 66744 TPM in leaves, and from 0.01 (actually 0, only for normalization) to 22303 TPM in roots, and expression levels of miR166 family members ranged from from 48 to 16705 TPM in leaves, and from 12 to 20693 TPM in roots (Table 3). Overall, a diverse range of responses was observed among treatments, tissues and family members, but variation between leaves and roots appeared to be more prevalent in less-conserved than in conserved miRNAs (Tables 3, 4 and 5).

### Identification of P-responsive miRNAs

**Figure 3:**
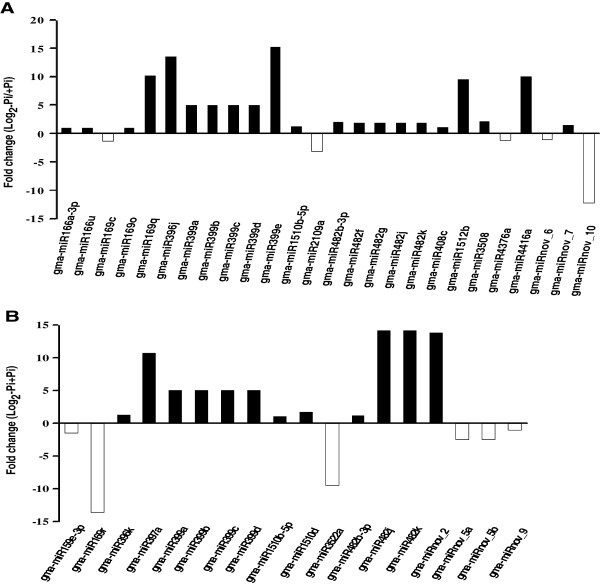
**Fold change (log**_**2**_**(-Pi/+Pi)) of miRNA expression analysis using transcript per million (TPM) transformed data from P treatment libraries.A**: miRNAs from leaves; **B**: miRNAs from roots. Black and white bar indicates -Pi-induced and -Pi-repressed miRNAs, respectively.

Soybean P-responsive miRNAs were identified by calculating the log fold change in read counts, log _2_(-Pi/+Pi). If this value was greater than 1, the miRNAs were considered to be induced by P deficiency. Figure 3A shows that the expression of 21 out of 126 (16.7%) mature miRNAs was stimulated in leaves by Pi starvation with fold changes ranging from 1 to 15.27. Moreover, in contrast to other -Pi-induced miRNAs, miR169q, miR396j, miR399e, and miR4416a were sharply induced in leaves by Pi limitation, with no expression detected in +Pi and expression over 10 in -Pi (Figure 3A; Tables 3 and 4).

**Figure 4:**
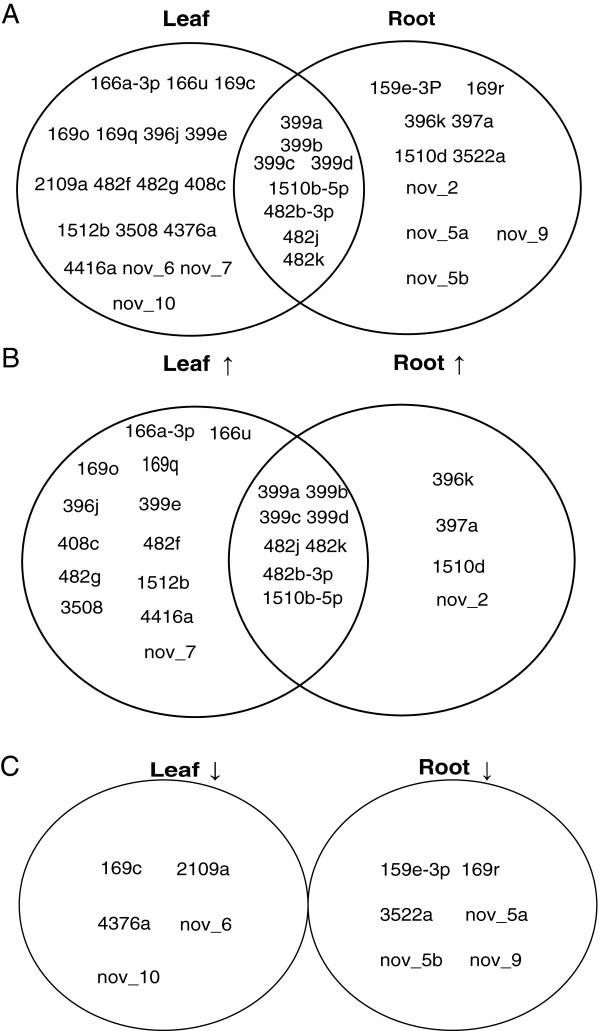
**Unique and overlapping soybean P-responsive miRNAs in leaves and roots induced or repressed by -Pi treatment.A**: Unique and overlapping soybean P-responsive miRNAs in leaves and roots; **B**: Unique and overlapping soybean miRNAs in leaves and roots up-regulated by -Pi treatment; **C**: Unique and overlapping soybean miRNAs in leaves and roots down-regulated by -Pi treatment.

In roots, twelve out of 126 (9.5%) miRNAs were stimulated by Pi starvation with log fold changes ranging from 1.03 to 14.18 (Figure 3B). The levels of miR397a, miR482j, miR482k, and miRnov_2 in roots were induced from no detected expression to considerable expression in +Pi and -Pi (Figure 3B; Tables 3 and 5). On the other hand, miR169r and miR3522a were significantly down-regulated by Pi depletion (Figure 3B; Tables 3 and 4). Figure 4A shows that thirty-six miRNAs were Pi-responsive in soybean genome-wide, with 26 and 18 being responsive in leaves and roots, respectively. Eight miRNAs were induced under -Pi conditions both in leaves and roots and none were repressed (Figure 4B, C; Tables 3, 4 and 5;). Perhaps more intriguingly, 5 miRNAs were tissue specific and had expression altered by P treatment. Those that were induced in -Pi relative to +Pi are the leaf specific miR396j and miRnov_7, and the root-specific miRnov_2 (Figure 4B; Tables 3 and 5). Those that were repressed are the leaf-specific miR4376a and the root-specific miR169r (Figure 4C; Tables 3 and 4). This tissue specificity and P responsiveness implies the tissue-specific roles of miRNAs in P signaling.

**Figure 5:**
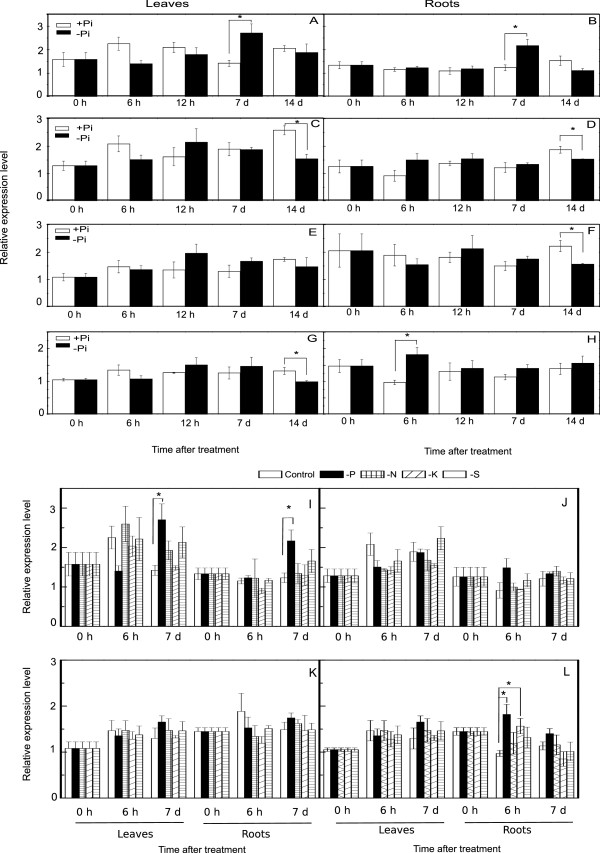
**Expression analysis of miRNA399a/b/c/d, miRnov_6, miRnov_9, miRnov_10 under Pi-starvation and others different nutrient deficiency. A-H**: expression patterns of miRNA399a/b/c/d/ (**A** and **B**), miRnov_6 (**C** and **D**), miRnov_9 (**E** and **F**), and miRnov_10 (**G** and **H**) in leaves and roots in +Pi and -Pi; I-L: expression patterns of miRNA399a/b/c/d (**I**), miRnov_6 (**J**), miRnov_9 (**K**), and miRnov_10 (**L**) in leaves and roots under different nutrient deficiency conditions. Stars above bar indicate the significant difference.

The expression levels of mature miR399, miRnov_6, miRnov_9, and miRnov_10 were determined through stem-loop real-time PCR [25]. Because mature sequences of miR399a, 399b, 399c, and miR399d are 100% identical (Table 3), it is impossible to distinguish the levels of the four mature miR399s with stem-loop PCR. The total abundance of miR399a/b/c/d was significantly induced by Pi starvation in both leaves (Figure 5A) and roots (Figure 5B) after 7 d of Pi starvation, but not at any other tested time point. The expression level of miRnov_6 in leaves (Figure 5C) and roots (Figure 5D) was decreased on day 14 of Pi starvation (P <0.05). The expression of miRnov_9 was repressed by P deficiency on day 14 in roots, but not in leaves (Figure 5E and F). Interestingly, the level of miRnov_10 was repressed in leaves on day 14 (Figure 5G) and induced in roots at 6 h (Figure 5H), which might indicate that miRnov_10 plays an important role in local and systematic P signaling pathways.

To determine whether the responses of the above-mentioned miRNAs are specific to Pi stress, the responses to N, potassium (K), and S starvation were explored. Expression alterations of marker genes *GmNRT2(Glyma13g39850)*, *GmHAK1*(*Glyma19g45260*), *GmSult1 (Glyma06g111500)* indicated that the tested soybean seedlings were really subjected to N, K, and S starvation, respectively (data not shown). The miRNA miR399a/b/c/d responded to Pi starvation in leaves and roots, but not to other nutrient deficiencies (Figure 5I). Neither miRnov_6 nor miRnov_9 responded to any nutrient deficiencies at 6 h and 7 day either in leaves or roots (Figure 5J, K), which is consistent with the requirement of 14-day Pi starvation for a response (Figure 5C, D, E, F). Therefore, we could not determine whether miRnov_6 or miRnov_9 was specifically responsive to Pi. The expression of miRnov_10 in roots was induced by Pi starvation and K deficiency at 6 h (Figure 5L), which was consistent with the previous test and indicative of a nonspecific response.

*AtIPS1* attenuates miR399s activity via the formation of the three-nucleotide bulge in the highly complementary region where cleavage occurs [26]. Upon searching in soybean transcriptome ( http://www.tigr.org), four *GmIPS* (*GmIPS1-4*) members were found. Among them, *GmIPS1* nearly perfectly matched miR399a, b, c, d, and e over the center region, thus forming a three-nucleotide bulge (Additional file 5), and thereby implying that soybean miR399 activity might be negatively modulated by *GmIPS1* as in Arabidopsis.

### Soybean root RNA degradome library sequencing

To determine the targets of soybean miRNAs, an LPR small RNA degradome library was constructed based on described methods [20]. A total of 28, 557,354 high quality reads containing more than 25 million clean reads were obtained from the root degradome library (Additional file 6). In addition, slightly more 21- than 20-nt sequences were obtained (data not shown). After excluding reads smaller than 18 nt and adaptors, approximately 88% of clean reads were analyzed (Additional file 6). Furthermore 83.93% of clean reads (21,185,857 out of 25,241,382) and 67.72% of unique reads (2,406,610 out of 3,553,894) were mapped to the soybean genome (Additional file 6).

### Target prediction of *Glycinemax* conserved, less-conserved, and novel miRNAs

Pairfinder was employed to analyze the dedradome sequencing data as described in Methods. Degradome data showed that 51 genes were the targets of 19 conserved miRNAs (Additional file 7), 10 genes were the targets of 4 less-conserved miRNAs (Additional file 8), and 11 genes were the targets of 8 novel miRNAs (Additional file 9). One gene, *Glyma08g21620* was detected to be the target of both miR166g and miRnov_2. Hence, a total of 71 genes were determined to be the targets of 126 miRNAs by degradome sequencing.

Since some miRNA targets were not detected in degradome sequencing, psRNAtarget ( http://plantgrn.noble.org/psRNATarget/) was employed as a complementary approach to predict miRNA target genes. In combination with degradome sequencing, a total of 154 genes were predicted or detected to be the target of 126 miRNAs. Putatively, 98 genes were attacked by 89 conserved miRNAs, 37 genes were the targets of 25 less-conserved miRNAs, and 20 genes were targeted by 12 novel miRNAs. Previous studies indicated that conserved miRNAs attach to targets in the CDS. Consistent with this, for the conserved and less-conserved miRNAs, 94.8% (182 out of 192) and 85.2% (40 out of 47) of cleavage sites were in CDS regions, respectively. For novel miRNAs, the percentage was 100%. Interestingly, the 28 transcripts targeted by the 12 novel soybean miRNAs included 19 transcripts for 7 miRNAs (miRnov_1a, miRnov_1b, miRnov_2, miRnov_3, miRnov_5a, miRnov_5b, miRnov_6, miRnov_7) in the root degradome library, and 9 transcript targets for four miRNAs (miRnov_4, miRnov_8, miRnov_9, miRnov_10) predicted through computational analysis (Additional file 9).

As for the conserved miRNAs, the number of targets for conserved miRNA (2.31 targets per miRNA) was higher than that of less-conserved miRNA (1.53 targets per miRNA) (Additional files 7 and 8). Among the conserved miRNAs, five (miR162c, miR166h-3p, miR166j-3p, miR390b,and miR408b-5p) were identified to have only one target, while the rest have two or more targets, most notably miR156d, miR167e, miR167g, miR167j, miR167k, miR172b-3p, and miR172h-5p (Additional file 7). In contrast, 15 out of 25 less-conserved miRNAs had only one target. However, the previously unreported less-conserved miR1508e and miR3508 each have seven targets (Additional file 8). *Glyma08g21610* and *Glyma16g34300*, which encode a AGO proteinendoing and a HD-ZIP protein respectively, were predicted target of miR166g and miR168 (Additional file 7). A NF-YA gene *Glyma10g10240* was the putative target of miR169c (Additional file 7). Importantly, these predicted cleavage sites for the three targets are consistent with previous RLM-5’ RACE results [20,27,28], supporting the reliability of our computational analysis.

In general, miRNAs that are conserved across plants, such as miR156, miR164, miR167 and miR169, target transcription factors (TFs), whereas less-conserved miRNAs target fewer TFs (Additional file 8). Overall, 54% (53 out of 98) of conserved miRNA target genes were TFs (Additional file 7), while only 2.7% (1 out of 37) and 10.0% (2 out of 20) of less-conserved and novel miRNA target genes, respectively, were TFs (Additional files 8 and 9). This is in accordance with previous study [20].

In regards to nutrient stress, the targets of root P-responsive miRNAs and their cleavage sites are highlighted in Table 6. A PHO2, two Pi transporter transcripts (*Glyma10g04230* and *Glyma14g36650*), and an AP2 protein gene (*Glyma01g13410*) were predicted to be targets of miR399a, b, c, d, and e (Table 6). In fact, miR2111 was detected in small RNA libraries in this study, but the level of it was too low to be filtered out based on the strict criteria. The target of miR2111 was predicted. As in Arabidopsis, a kelch repeat-containing F-box protein, *Glyma16g01060*, was the putative target of miR2111. The targets of the Pi starvation-induced miR169o, miR397a, miR408c, miR4416a, miRnov_2, and miRnov_7 were detected in the degradome library or computationally predicted, as were the targets of the P limitation-repressed miRNAs miR159e-3p, miR169r, miR2109, miR4376, miRnov_5a, miRnov_5b, and miRnov_6a (Table 6). It must be pointed out that most of the targets listed in Additional files 7, 8 and 9 need to be experimentally confirmed with RLM-5’ RACE or transit expression analysis.

**Table 6:** Target genes of P-responsive miRNAs in soybean

**miRNA**	**Target gene**	**Target description**	**Cleavage site (nt)**	**Abundance**	**Target site location**
				**(TP10M)**	
**UP-regulated miRNAs by -Pi**					
m	Glyma20g37690.1	No annotation^c^	2526		CDS
miR166u	Glyma05g06070.1	Myb^b^	1806		CDS
miR169o	Glyma06g03940.1	Peptidase S24-like^a^	584	38.429	CDS
miR169q	Glyma18g07890.1	NF-YA^b^	949		CDS
miR396j	Glyma02g45340.1	LRR/NB-ARC domain/TIR domain^b^	710		CDS
	Glyma10g00320.1	Protease family S9B^b^	1252		CDS
	Glyma12g28570.1	Core-2/I-Branching enzyme^b^	753		CDS
	Glyma16g00260.1	Core-2/I-Branching enzyme^b^	711		CDS
	Glyma17g35500.1	Syntaxin 6, N-terminal^b^	988		CDS
miR396k	Glyma12g01390.1	Cytokinin dehydrogenase^c^	730		CDS
miR397a	Glyma11g19150.1	Male sterility protein^a^	1484	34.8634	CDS
	Glyma12g09270.1	Male sterility protein^a^	1475	34.8634	CDS
miR399a/b/c/d	Glyma10g04230.1	Phosphate transporter^b^	407		CDS
	Glyma01g13410.1	AP2^b^	27		CDS
	Glyma14g36650.1	Phosphate transporter^b^	240		CDS
	Glyma13g31290.1	PHO2^b^	1050		5’UTR
miR399e	Glyma10g04230.1	Phosphate transporter^c^	407		CDS
	Glyma13g31290.1	PHO2^c^	1050		5’UTR
miR408c	Glyma06g12680.1	Plastocyanin-like domain^a^	207	55.8606	CDS
	Glyma08g13510.1	Plastocyanin-like domain^a^	198	18.224	CDS
miR1510b-5p	Glyma10g16090.1	PCI domain/eIF3 subunit 6 N terminal domain^c^	1451		3’UTR
miR1510d	Glyma11g04630.1	Domain of unknown function (DUF296)^c^	1152		3’UTR
miR482f	Glyma05g01650.1	PPR^c^	521		CDS
miR482g	Glyma05g01650.1	PPR^c^	521		CDS
miR482j	Glyma05g06070.1	Myb^b^	282		CDS
miR482k	Glyma05g06070.1	Myb^b^	282		CDS
miR482b-3p	Glyma09g39410.1	LRR/NB-ARC domain/leucine-rich repeat-containing protein^c^	513		CDS
miR1512b	Glyma08g17790.1	D-mannose binding lectin/Domain of unknown function (DUF3403)^b^	1209		CDS
miR3508	Glyma06g42170.1	Protein of unknown function (DUF_B2219)/Polyphenol oxidase middle domain^b^	1048		CDS
	Glyma07g31290.1	Protein of unknown function (DUF_B2220)/Polyphenol oxidase middle domain^b^	2569		CDS
	Glyma07g31310.1	Protein of unknown function (DUF_B2221)/Polyphenol oxidase middle domain^b^	1117		CDS
	Glyma13g25150.1	Polyphenol oxidase middle domain/Common central domain of tyrosinase^b^	1096		CDS
	Glyma13g25180.1	Protein of unknown function (DUF_B2219)/Polyphenol oxidase middle domain^b^	1025		CDS
	Glyma13g25260.1	Protein of unknown function (DUF_B2220)/Polyphenol oxidase middle domain^b^	1199		CDS
	Glyma15g07710.1	Protein of unknown function (DUF_B2221)/Polyphenol oxidase middle domain^b^	1077		CDS
miR4416a	Glyma10g06650.1	Uncharacterized nodulin-like protein^b^	1544		CDS
miRnov_2	Glyma08g21620.1	START domain^a^	2712	19.0164	CDS
	Glyma08g21620.2	START domain^a^	1892	13.8661	CDS
	Glyma18g51750.1	LRR/NB-ARC domain^a^	1840	46.3525	CDS
miRnov_7	Glyma19g37520.1	Enolasea	755	904.8633	CDS
**Down-regulated miRNAs by -Pi**					
miR159e-3p	Glyma13g25716.1	Myb^a^	1340	97.459	CDS
	Glyma15g35860.1	Myb^a^	937	198.4836	CDS
miR169c	Glyma10g10240.1	NF-YA^c,d^	1174		3’UTR
miR169r	Glyma18g07890.1	NF-YA^b^	949		CDS
miR2109	Glyma16g29650.1	Heavy-metal-associated domain^a^	297	56.653	CDS
	Glyma16g33950.1	Leucine-rich repeat-containing protein^a^	86	25.3552	CDS
	Glyma16g33980.1	Plant basic secretory protein^a^	833	51.8989	CDS
	Glyma16g34060.1	transmembrane receptor activity^a^	185	28.9208	CDS
	Glyma16g34060.2	transmembrane receptor activity^a^	185	28.9208	CDS
miR3522	Glyma07g31270.1	Protein of unknown function (DUF_B2219)/Polyphenol oxidase middle domain^c^	418		CDS
miR4376a	Glyma09g36420.1	F-box/LRR^c^	1297		
miRnov_5a	Glyma06g08730.1	No annotation^a^	681	60.2186	CDS
	Glyma06g08730.2	No annotation^a^	926	60.2186	CDS
	Glyma06g08730.4	No annotation^a^	968	318.9207	CDS
miRnov_5b	Glyma06g08730.1	No annotation^a^	681	60.2186	CDS
	Glyma06g08730.2	No annotation^a^	926	60.2186	CDS
	Glyma06g08730.4	No annotation^a^	968	318.9207	CDS
miRnov_6a	Glyma05g07780.1	DEAD/DEAH box helicase^a^	1019	19.8087	CDS
	Glyma06g23290.1	DEAD/DEAH box helicase^a^	1036	19.8087	CDS
	Glyma14g40680.1	EamA-like transporter family^a^	818	72.1038	CDS
	Glyma17g13230.1	DEAD/DEAH box helicase^a^	268	47.9372	CDS
	Glyma18g22940.1	DEAD/DEAH box helicase^a^	1005	19.8087	CDS
miRnov_9	Glyma05g00880.1	Sensor histidine kinase-related^b^	1165		CDS
	Glyma04g39860.1	Peroxidase^b^	260		CDS
	Glyma06g15030.1	Peroxidase^b^	256		CDS
miRnov_10	Glyma08g20840.1	Dof domain, zinc finger^b^	1001		CDS
	Glyma20g26290.1	Cyclinb	895		CDS
	Glyma10g40990.1	Cyclinb	626		CDS
	Glyma13g17410.1	CAAX amino terminal protease family alpha/beta hydrolase domain containing^b^	3678		CDS

The potential targets of soybean P-responsive miRNAs in leaves and roots were predicted by degradome sequencing and computationally using Pairfinder and psRNAtarget. The *Glycine max* transcriptome is available in Phytozome ( http://www.phytozome.org). For conserved miRNAs, homology of which can be found in other plant species apart from legume; for less-conserved miRNAs, homology of which can be found in legume (legume-specific); for novel miRNAs, which were not previously in miRBase and not assigned into any family were denoted by miRnov (soybean-specific).

^a^: predicted from degradome;

^b^: predicted with Pairfinder developed by BGI ( http://www.bgi.cn);

^c^: predicted from psRNAtarget ( http://plantgrn.noble.org/psRNATarget/?function=3);

^d^: experimentally confirmed target [20,27,28].

AP2, AP2 domain protein; CDS: coding sequence; Cleavage site: nucleotide position in cDNA cleaved by miRNA from 5’ to 3’; Myb, Myb family of transcription factors; NF-Y, NUCLEAR FACTOR Y (also CCAAT, CCAAT-binding transcription factor); TP10M: transcripts per 10 million reads; UTR: untranslated region.

### Determination of targets of miR399, miR2111, and miR159e-3p through RLM-5’ RACE

**Figure 6:**
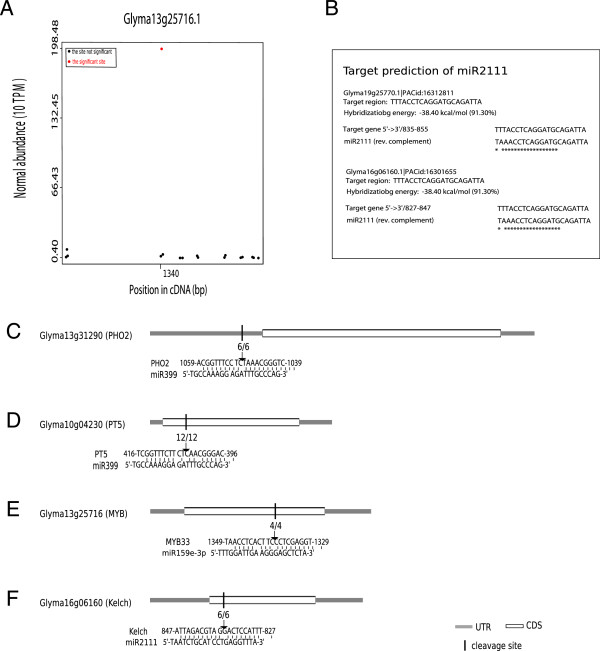
**Confirmation of the targets of miR399, miR2111, and miR159e-3p by RLM-5’ RACE.** Degradome sequencing analysis indicating a cleavage site at position 1050 from the 5’ end of the transcript of *Glyma13g25716.1* is targeted by miR159e-3p (**A**). Target prediction of miR2111 with WMD3 (http://www.weigelworld.com) showing *Glyma19g25770* and *Glyma16g06160* as putative targets of miR2111 (**B**). Cleavage sites from the 5’ end for Glyma13g31290 (PHO2-1) by miR399 (**C**), *GmPT5* by miR399 (**D**), *Glyma13g25716* by miR159e-3p (**E**), and *Glyma16g06160* by miR2111 (**F**). The arrow indicates the cleavage position in the transcript of target genes, and the number above the arrow shows the sequenced clone numbers of PCR products.

To test the accuracy of target gene cleavage site location results, RLM-5’ RACE was compared with predictions. The cleavage site of miR399 in PHO2 is predicted to occur at position 1050, and in *Glyma10g04230* at position 407 (Additional file 7). Figure 6A shows the putative cleavage site of miR159e-3p in the target gene *Glyma13g25716* at position 1340 as determined through degradome sequencing. In addition, Figure 6B shows the cleavage sites of miR2111 in two target genes, *Glyma19g25770* and *Glyma16g06160*, as predicted with WMD3 ( http://www.weigelword.com). RLM-5’ RACE has been successfully employed to determine cleavage sites of miRNAs in soybean [20,27]. In this study, RLM-5’RACE using RNA from Pi-depleted roots confirmed the cleaved fragments of *GmPHO2* (*Glyma13g31290*) and *GmPT5* (*Glyma10g04230*) mRNA predicted previously (Figure 6C, D). The experimentally determined cleavage site of miR159e-3p in *Glyma13g25716* (Figure 6E) matched the predicted site, and the cleavage site in *Glyma16g06160* predicted with WMD3 (Figure 6F). These results indicate that degradome sequencing and computational predictions can reliably predict miRNA interactions with target genes.

### Possible functions of soybean miRNAs’ targets

A total of 154 target genes were identified through degradome sequencing and computational predictions (Additional files 7, 8 and 9). To better understand the biological functions of these genes in soybean, GO analysis [29] was employed to classify target genes based on their involved biological processes.

As shown in Additional file 10, a total of 154 target genes are positively or negatively involved in many biological processes in soybean. For instance, target genes positively regulate the following processes: (1) nucleoside, nucleotide, and nucleic acid metabolic process (45/154, GO:0019219); (2) RNA metabolism (35/154, GO:0051252); (3) gene expression (49/154, GO:0010468); (4) macromolecule biosynthetic process (46/154, GO:0010556); (5) meristem development (10/154, GO:0048509). On the other hand, target genes negative regulate these processes: (i) seed development (31/154, GO:0048316); (ii)shoot development (25/154, GO:0048367);(iii) root development (19/154, GO:0048364); (iv) meristem initiation (10/154, GO:0010014). These results indicate the important roles of target genes in soybean in response to Pi starvation.

**Figure 7:**
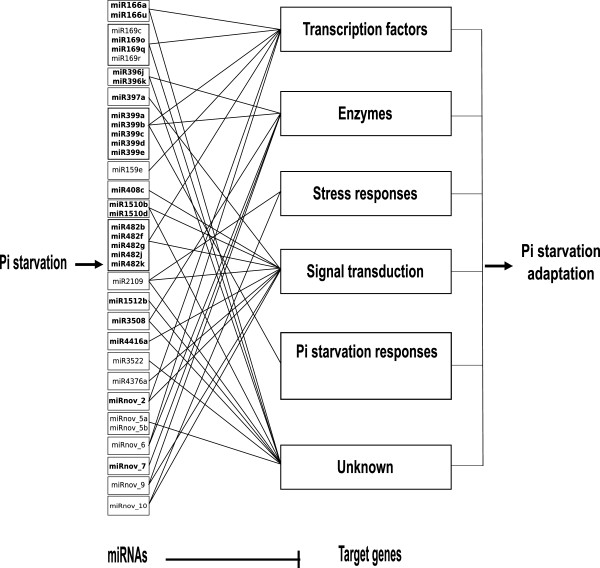
**Possible functional networks for P-depletion responsive miRNAs in soybean.** Relationships between 25 P-depletion induced and 11 P-depletion repressed miRNAs and their target genes shown based upon putative physiological functions. Bold font and normal font indicate -Pi induced or repressed miRNAs, respectively.

A more narrow focus on the targets of -Pi induced and repressed miRNAs returns functions that can be grouped into several categories based on the involved processes (Table 6). These include: (i) protein synthesis or degradation; (ii) P uptake and transport; (iii) stress-related processes; (iv) cell division; and (v) ROS homeostasis. These results indicate that complex networks of P signaling are present in soybean. Figure 7 outlines the possible functions of P-responsive miRNAs and their target genes.

### *cis*-element analysis in the promoter of P-responsive miRNAs

AtPHR1 binds the promoter region of miR399 in Arabidopsis [30], indicating control of miRNA expression by TFs. *cis*-element analysis shows that TATA-box and TSS elements in the pre-miRNA upstream region can be found in over 90% of miRNA genes (Additional file 11). A total of 270 TSSs and 230 TATA-boxes were found in the promoters of 126 soybean miRNA genes (Additional file 11), with more TSSs and TATA-boxes found within 1.0 kb upstream of pre-miRNA than in the 1.0 to 2.0 kb upstream region (Additional file 12).

**Figure 8:**
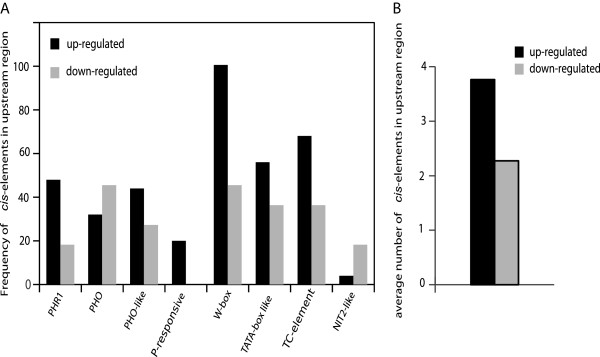
**Frequency of P-responsive *cis*-elements in the promoter regions of P-responsive miRNAs.** Frequency of *cis*-elements types (**A**), and average number of *cis*-elements per upstream region (**B**) from 25 up-regulated and 11 down-regulated miRNA genes differentially expressed in -Pi relative to +Pi.

Several well-defined Pi-responsive *cis*-elements were selected as references to identify P-responsive *cis*-elements potentially regulating the currently studied miRNAs [31]. A total of 377 Pi-responsive elements were detected for 126 miRNA genes, the average number of *cis*-elements was 3.02 (Additional file 13). Among them, miR156w, miR156x, miR166g, miR168c and miR168d all contained 8 *cis*-elements, and miR166a-5p harbored nine. Genes for miR399a, miR399b, miR399d and miR399e had PHR1 binding sites, but miR399c only had a W-BOX binding site (Additional file 13), indicating the expression of miR399a, b, d, and e might be regulated by PHR1 in soybean. The average frequency of PHR1, PHO-like, Pi-responsive, W-box, TATA-box, and TC elements in -Pi-induced miRNA promoter regions was higher than that in the -Pi-depressed miRNA promoter region, while the frequency of PHO and NIT2 elements was lower in the Pi-induced miRNA promoter regions than that in the -Pi- depressed miRNA promoter regions (Figure 8A).

## Discussion

### Deep sequencing of small RNAs and RNA degradome in soybean

Soybean is an important crop that provides oils and proteins to human and animals. Little is known about the involvement of miRNAs in soybean leaf and root development, and P signaling. Although high-throughput sequencing has been employed to reveal soybean miRNAs [20], there are likely many miRNAs to still be found. SBS has been successfully employed to find miRNAs genome-wide in rice [32] and *Glycine max*[20]. Figure 1 shows a major peak at 21 nt in the total small RNAs from four small RNA libraries. A peak at 19 nt found in Arabidopsis root libraries [10] was not found in the current study. On the other hand, our data identify 24 nt unique small RNA species as dominant over other kinds of small RNAs in four small RNA libraries, which is consistent with previous studies [10,20,23]. One possible reason for the prevalence of 24 nt small RNAs is that most precursors of siRNAs are processed into 24 nt-long siRNAs by DCL3 [1]. In regards to nutrient effects, it has been reported that P availability does not change total small RNA profiles in rice leaf libraries [23]. This is consistent with the results reported here (Figure 1), but it does not address the fact that a number of individual miRNAs are differentially expressed among P treatments (Figures 2, 3 and 4). Hsieh et al. [10] (2009) reported that more than 24% of small RNA reads mapped to tRNA in the Arabidopsis genome in two root libraries, while the percentage in leaf libraries is lower [10]. This high percentage of tRNA in root libraries was not observed in soybean (Additional file 2). In conclusion, the profiles of total small RNA in plants are dependent on plant species, tissue type and environmental conditions.

Since 2008, degradome sequencing has been employed to screen Arabidopsis miRNA targets [33]. A total of 174 genes targeted by 87 unique miRNAs were identified in rice cultivar 93-11 from a young panicles degradome library [32]. Here, degradome sequencing detected 71 genes to be cleaved by conserved, less-conserved, and novel soybean miRNAs. RLM-5’ RACE has confirmed the targets of miR166g, miR168 and miR169c in previous work [20,27,28], as well as the targets of miR399, miR2111 and miR159e-3p in this study. Moreover, the cleavage sites were in accordance with predictions or degradome sequencing (Additional file 7). This indicates that degradome analysis is a powerful tool to find soybean miRNA targets. The limitation of this study is that just one degradome small RNA library was produced from Pi-starved roots. MicroRNA-degraded fragments existing specifically in leaves or other organs above the roots were not sequenced. Nevertheless, using a combination of experimental and computational approaches, 95 new targets for conserved, less-conserved, and novel miRNAs were putatively identified here. Accordingly, identification of more targets of soybean miRNAs will shed fresh light on the functions of soybean miRNAs in the near future.

### Discovery of conserved, less-conserved, and novel miRNAs in soybean

Presently, a total of 395 soybean mature miRNAs (362 precursors) were curated in miRBase 18.0. In this study, we adopted very strict criteria to determined candidate miRNAs as outlined above. Interestingly, in contrast to previous studies [20,34,35], 62 of the conserved miRNAs (Table 3), 18 of the less-conserved miRNAs (Table 4), and 12 of the novel soybean-specific miRNAs (Table 5) were new discoveries in soybean. A possible explanation for these discoveries is the sampling of tissues from multiple time points and treatments. RNA was sampled from leaves and roots at different time points and P treatments, which increases the odds of finding previously unreported miRNAs in soybean.

Our results significantly improve our understanding of miRNA families. The first to mention is the miR156 family, which is big and conserved across plants, including 12 miR156 members in both Arabidopsis and rice, 11 members in maize, and 8 in *Medicago* ( http://www.phytozome.org). Here, the soybean miR156 family members have been expanded from 15 to 29 (Table 3). In addition, 11 miR166s were identified for the first time (Table 3), indicating that miR166 is also a very big miRNA family in soybean. We note that as an ancient polyploidy descendent, the genome of *Glycine max* was duplicated two times, 59 and 13 million years ago [36]. These events likely led to significant increases in the two miRNA families listed above, as well as, potentially increasing membership in other families. Furthermore, miR1507, miR1509, and miR1510 were identified, and to date, have only been found in *Medicago* and soybean (Table 2). In short, the known sizes of soybean miRNA families were significantly expanded through this study. However, we did not detect miR157, miR393,miR395, or miR398 as previously reported. Possible explanations are that (i) strict criteria were implemented in this study for identification of mature miRNAs; and (ii) the RNA samples were limited to low P-stressed leaves and roots, along with their control, so -S-induced miR395 and -Cu-induced miR398 were not likely to be detected.

### miRNAs in soybean leaves and roots

MiRNAs play crucial roles in leaf and root development [1]. Figure 2 shows 112 miRNAs detected both in leaves and roots, along with leaf- and root-specific miRNAs extracted from +Pi or Pi treatments. Comparable to the reported very high levels of miR156f and miR156h in soybean leaves [35] were the currently reported very high expression levels of miR156d, miR156h, and miR156o in soybean leaves (Table 3). This implies crucial roles for all of these miRNAs in leaf development. The high expression of 11 miRNAs (gma-miR164, miR167, miR168b, miR319a, miR396a, miR482b, miR482b*, miR2118a, miR2118b, miR1508a, and miR1509a) in soybean leaves has been verified by microarray analysis, as were low expression levels of miR169a, miR390c, miR1507c, and miR1510a [35]. In this study, the abundance of several miR169 family members (miR169c, miR169p, miR169q, and miR169r) was low yet still detectable in soybean leaves (Table 3), which indicates some roles for these miRNAs in leaves, and stands in contrast to the report that gma-miR169 is abundant in soybean shoot apical meristem (SAM) and undetectable in mature soybean leaves [35]. The higher expression of miR164 in mature soybean leaves than in SAM has also been reported [35], and consistent with this, the levels of miR164e, f, g, h, and i in leaves were much higher than in roots (Table 3). Over-expression of rice miR172 results in loss of spikelet determinacy and flower abnormalities [37]. Interestingly, miR172b-3p, miR172h-5p, and miR172k were present at higher levels in leaves than in roots (Table 3), conversely, miR172b-3p was still found in significant numbers in roots. Transcripts of miR396k in leaves were higher than that in roots. This miRNA targets the cytokinin (CTK) oxidase/dehydrogenase encoded by *Glyma12g01390* (Additional file 7). As CTK regulates meristem activity in leaves [38], miR396k appears to regulate soybean leaf meristem activity. One interesting study that this work brings to mind is the exploration of the roles of the 10 and 12 leaf-specific miRNAs under +Pi and -Pi conditions, respectively (Figure 2) in soybean compound leaf development.

Many miRNAs have been reported to regulate root development [13,39,40]. The expression of miR160-resistant AUXIN RESPONSE FACTOR 17 (ARF17) leads to a shorter primary root and few lateral roots in Arabidopsis [39]. Ata-miR164 mediates lateral root development through attacking NAC1, and miR167 modulates adventitious rooting via targeting ARF6 and ARF8 [40]. Auxin receptors TIR1, AFB1, AFB2, and AFB3 are targets of miR393 [13]. Relative to other miR166s, gma-miR166g, miR166k, miR166h-3p and miR166j-3p were abundant in soybean roots (Table 3). AUX/IAAs are cleaved by miR167s (Additional file 7), and the levels of miR167e, miR167j, and miR167l are high in roots (over 400 TPM) (Table 3). Under +Pi conditions, miR169q, miR169r, miR4416a and miR1512b were specifically expressed in roots (Tables 3 and 4), and under -Pi conditions, miRnov_2 was specifically induced in roots (Table 5), indicating these miRNA are crucial regulators in root development and adaptations to P starvation. It was reported that five conserved miRNAs (miR159, miR162, miR166, miR390, and miR399) presented similar expression levels in root apexes and nodules, but miR169, miR171, miR393, and miR396 enriched in root tips [41]. However, few miRNAs were reported to control root development and nodulation in soybean, future studies should focus on the field. All together, our results demonstrate that miRNAs are intimately involved in many aspects of plant development, and much of these roles remain to be elucidated.

### P-responsive miRNAs in soybean leaves and roots

In this study, we found 26 P-responsive miRNAs in leaves and 18 P-responsive miRNAs in roots (Figure 4A). Recently, more P-responsive miRNAs have been found in Arabidopsis [10], common bean [42], and white lupin [43]. MicroRNA chip experiments showed that eight miRNAs (miR156/157, miR167, miR168, miR319, miR159, miR894, miR1507, and miR1509) were induced by Pi starvation in soybean leaves, and seven miRNAs (miR159, miR894, miR1507, miR1509, miR396, miR474, and miR482) were induced in soybean roots by low P [31]. In Arabidopsis roots, miR156a, b, c, d, e, and f are moderately induced by P deficiency, and the ata-miR156 family is also induced by -N, -K [10]. In this study, no miR156 was found to be induced by low P in leaves or roots (Table 3). A possible reason is that RNA samples were pooled over multiple time points spanning short to long durations of stress. Hsieh et al. [10] (2009) extracted RNA from Arabidopsis roots and leaves treated for 7 days with low P [10].

In Arabidopsis, miR399s induced by Pi starvation are transported over long distances through phloem [11,30]. The expression of miR399 has been predominantly found in vascular tissues, especially in root phloem companion cells in Arabidopsis [12]. MiR399 accumulated in *Medicago* and tobacco roots during arbuscular mycorhizal symbiosis [44], and targeted a putative phosphate transporter (*Mendtr5g076920.1*) [45]. Concordantly, soybean miR399s (miR399a, miR399b, miR399c, and miR399d) were induced by Pi starvation both in leaves and roots, but the levels in leaves are around 2.5 times higher than in roots (Table 3). Whether they are only accumulated in leaves and later transported to roots via the phloem, or if they play active roles in shoot P transport remains an open question. Grafting experiments involving root stocks and shoots with divergent miR399 sequences or expression patterns might answer this question. The qRT-PCR data in the present study confirms that miR399a/b/c/d specifically responds to Pi deprivation (Figure 5), while RLM-5’ RACE confirms that soybean miR399 cleaves *PHO2* and *GmPT5*, as in Arabidopsis, rice, and common bean [42]. It is worth exploring the conservation and variation of miR399s and target genes between soybean and other plant species.

Soybean miR396k and miR397a were also added as -Pi induced miRNAs in this study (Table 3). Arabidopsis miR398a, miR398b, miR398c, and miR408 were repressed in leaves under Pi-depleted conditions [10], while Cu depletion induced the expression of miR408, miR399, and miR2111 in Arabidopsis, rice and Brassica [12]. In this study, miR408c was stimulated by low P in soybean leaves (Table 3, Figure 3), indicating divergent roles for this family among plant species, or complex interactions between P and Cu signaling in plants that remains to be adequately outlined. In contrast to miR482 expression in Arabidopsis [10], five miR482s were found to be induced by -Pi treatment (Table 3; Figure 3). Additionally, miR778 and miR827 are reported to be specifically induced by -Pi in Arabidopsis [10], but neither was detected under -Pi conditions in soybean. To date, no miR827 has been reported in legumes ( http://www.mirbase.org), which, together with the present results, indicates that the miR827 gene may have ceased to function or has dramatically diverged over the course of legume evolution.

Past studies suggested that only miR399 and miR395 are transported from shoots to roots via phloem but not xylem vessels [12,16]. With miRNAs only being found in phloem, and not xylem vessels [16], then it might be possible to differentiate local and systematic miRNA mediated responses of soybean to low P availability by profiling miRNAs in phloem sap in future.

A total of 125 putative *cis*-elements in 24 soybean-Pi responsive miRNA genes were found and P-responsive motifs exist in the promoter regions of 54 non-phosphorus responsive miRNAs [31]. Interestingly, the total P-responsive element frequency in P-responsive miRNAs was higher than that in non-responsive miRNAs (Figure 8B). Whether this difference in *cis*-element frequency is the direct reason for induced expression of P-responsive miRNA under P stress in soybean is, potentially a worthwhile and enlightening project.

### Possible functions of miRNAs/target modules in soybean

Many miRNAs function in growth, development and stress adaptations through the regulation of TFs [2]. Consistent with this, a total of 51 TF genes were targeted by conserved (Additional file 7) and less-conserved miRNAs (Additional file 8), indicating crucial roles for these miRNAs in soybean.

Fifteen miR156s target *SPL*s in Arabidopsis [46]. Among these, *SPL3*, *SPL4*, and *SPL*5 function in controlling flower time and phase change, while *SPL9* and *SPL15* play roles in leaf initiation. Over-expression of *SPL3* and *SPL9* stimulates flowering and over-expression of miR156 delays flowering via down-regulation of SPL activity [47]. In *Glycine max*, there are 45 genes encoding SPL proteins ( http://www.phytozome.org). In this study, 16 *SPL* transcripts are targets for 14 miR156 family members (Additional file 7). This data also reveals that 6 soybean CUC-like genes are targets of miR164 (Additional file 7), suggesting the functions of miR164/CUC modules in soybean leaf development and growth as they do in Arabidopsis [48].

Several HD-ZIP class III genes such as *PHV*, *REV*, and *AtHB15* are negatively regulated by miR165/166 [49]. Consistently, four HD-ZIP transcription factors were predicted to be cleaved by miR166 family miRNAs (Additional file 7). NF-YAs, the targets of miR169, participate in N and drought stress responses [50]. Soybean 5 NF-YA transcripts are potentially targeted by miR169 members (Additional file 7). In addition, miR169c and miR169r were down-regulated by -Pi in leaves and roots respectively, and miR169q was up-regulated in leaves by low P (Table 6). Hence, miR169s might act as integrators of P and N signaling. Ata-miR169s are down-regulated by low P, consistently,some of which have PHR1 binding sites in the upstream region [10]. PHO-like binding sites and/or W-box elements were detected in promoter regions of soybean 169c, 169o, and 169q (Additional file 12). *BAM3* (*At4g20270*) encodes a receptor kinase-like protein that functions in shoot and flower meristem development [51]. Soybean miR390b, d, e and f all target *Glyma02g45010*, which shows very high similarity with *BAM3*.

In contrast to miR399 activity in Arabidopsis and rice [10], soybean miR399 targets PHO2 in the 5’ UTR but not in the coding region (Figure 6C). GmPT5 (Glyma10g04230) was reported to be responsible for P homeostasis in nodule development [52]. RLM-5’ RACE data verified predictions that miR399a/b/c/d/e cleaves GmPT5 (Additional file 7, Figure 6D), implying potential regulation of P nutrition in nodules by miR399. Moreover, *GmIPS1* might act as a RNA mimic to attenuate miR399 activity as *AtIPS1/At4* does in Arabidopsis [26] (Additional file 5). Then RNA mimcry might be a useful tool to decipher other miRNA functions in soybean. Among those with unknown functions is soybean miR2111, which attacks a kelch repeat-domain containing F-box protein gene (Figure 6F), as it does in Arabidopsis [10]. However, the function of miR2111 and its target in Arabidopsis is still unclear. Exploring the role of miR399 and miR2111 in soybean in the near future at genetic and biochemical levels promises to yield useful insights into how these miRNAs function in plants, and their effects on associated networks.

*AtAGO1*(*At1g48410*) is cleaved by miR168a and miR168b in Arabidopsis [53]. *Glyma16g34300* and *Glyma09g29720*, homologues of *AtAGO1* are also targets of miR168 in soybean (Additional file 7). Pentatricopeptide repeat (PPR) proteins are predicted to be involved in RNA editing and metabolism in mitochondria and are essential for 5’end processing of transcripts [54]. MiR1508d targets one PPR transcript, while miR1508e targets 7 PPR transcripts (Additional file 8). In Arabidopsis, three PPR genes, *At1g06580*, *At1g62720*, and *At1g62670*, were predicted to be the targets of miR161 [55]. These results indicate that regulation of PPR by miRNAs is conserved across plants, though it may be accomplished by different miRNAs among plant species. In Arabidopsis, SGS3 is involved in trans-acting siRNAs generation, and thus participates in the post-transcriptional gene silencing and natural virus resistance [56]. Interestingly, miR2118a and miR2118b both target an X1-like transcription factor encoded by *Glyma05g33260*, which is a homologue of *AtSGS3*. OsBIRH1, a DEAD box RNA helicase, regulates defense responses [57]. Four DEAD helicase were predicted to be the targets of miRnov_6 (Table 6). Taken together, these results suggest that RNA metabolism is also tightly regulated by miRNAs in soybean.

Accumulation of reactive oxygen species (ROS) is one strategy for plants to cope with early stages of abiotic stress. However, higher ROS levels will damage proteins, nucleic acids, and lipids. P, K and Cu deficiency, as well as, drought stress boost ROS levels [58]. Under drought conditions, miR408 is strongly induced in photosynthetic tissues in *Medicago*[58]. Table 3 demonstrates that miR408c was highly expressed in leaves in +Pi and induced even further in -Pi. These results imply that miR408c might be an integrator of stresses. Superoxide dismutase (SOD), peroxidase (POD), glutathione S-transferase (GST), and cytochrome P450 genes are strongly induced by long-term Pi starvation [59], further supporting the notion that ROS participate in root responses to low P stress. Interestingly, POD, NADP oxidase, and cytochrome P450 genes were targets of miR1507a, miRnov_1, and miRnov_9 (Table 6; Additional files 8 and 9).

*Glyma17g05970.1* was targeted by miR4376-5p, and miR4376-5p was undetectable in roots (Additional file 8; Table 4). The homologue of Glyma17g05970.1 in Arabidopsis regulates root hair growth, trichome development, and organelle trafficking [60]. Loss of function of *AtENO1* (*At1g74030*), an Arabidopsis phosphoenolpyruvate enolase, results in distorted trichomes and fewer root hairs [60]. An enolase,*Glyma19g37520* was the target of gma-miRnov_7, which is also specifically expressed in leaves. In roots, the abundance of miR4416a is higher than in leaves, and its target is a nodulin-like protein gene, *Glyma10g06650* (Additional file 8). This implies that miR4416a is potentially involved in nodule development in interactions with rhizobial symbionts.

The cell division activity gradually decreases around the meristem, leading to the determinate growth of Arabidopsis [61]. P deprivation increases the expression of Cyclin D3 [62]. The data herein revealed that two Cyclin D3 genes were putative targets of gma-miRnov_10 (Table 6), and miRnov_10 was down-regulated in leaves stressed by -Pi (Figure 3; Table 5). Stunted growth of P stressed soybean might be mediated through the actions of miRnov_10.

Recently, 28 TFs in maize roots have been documented to be induced by P limitation, while 14 TFs are repressed [63]. Although putative P-responsive *cis*-elements were found in soybean P-responsive miRNA genes (Figure 8), the TFs responding to Pi starvation, and controlling root or leaf development in soybean remain unknown. In this study, many TFs were predicted to be targets of soybean miRNAs (Additional files 7, 8 and 9). Identifying and understanding the activities of those TFs will likely provide insight into how plant growth and development respond to P deficiency.

## Conclusions

A total of 126 miRNAs were identified in soybean through deep sequencing, including 92 previously unidentified (Tables 3, 4 and 5). Among these, leaf- and root-specific miRNAs were determined (Figure 2), and P-responsive miRNAs in leaves and roots were identified (Figure 4A). These 126 soybean miRNAs target 154 genes as revealed via degradome sequencing and computational predictions (Addititonal file 7, 8 and 9). Use of qRT-PCR verified the expression of four P-responsive miRNAs and 5’ RACE confirmed targets of miR399, miR2111, and miR159e-3p. Finally, *cis*-element analysis indicates the existence of P-responsive motifs in the promoter region of soybean miRNA genes. Taken together, these findings provide useful information for plant scientists to decipher soybean miRNA functions and establish a framework for exploring P signaling networks regulated by miRNAs. More extensive analysis of these miRNAs across time and spatial scales, and transgenic studies will facilitate exploring their roles in P signaling and leaf or root development.

## Methods

### Growth of soybean

The seeds of the sequenced soybean (*Glycine max* L. Merrill cv.Williams 82) were germinated in paper pouch for 2 days at 28°C in darkness and then grown in light for further 2 days, the uniform seedlings were transplanted into full nutrient solution (pH=5.9),which contained 250 *μ*M KH_2_*PO*_4_ and grown 5 days; soybean seedlings with the first fully developed trifoliate leaves were transferred into phosphate (Pi)-sufficient (250 *μ*M KH_2_*PO*_4_, +Pi) or Pi-deplete (0 *μ*M KH_2_*PO*_4_, -Pi) nutrient solution,respectively.Leaves and roots were separately sampled at 0 h, 6 h, 12 h, 7 day and 14 day after treatment. For nitrogen deprivation, NH_4_*NO*_3_ was omitted and KCl was substituted for KNO_3_. For potassium depletion, KNO_3_ was omitted. For sulfate starvation, all ${\text{SO}}_{4}^{2-}$ was substituted with Cl^-1^. All soybean seedlings were cultured in 24-l plastic boxes containing nutrient solution as indicated. The nutrient solution was automatically aerated 15 min every 3 h, and was replaced with fresh solutions every two days. Soybean plants were grown in a green house with 16 h light cycles at the Root Biology Center of South China Agriculture University.

### Determination of ratio of root to shoot and concentration of soluble phosphate (SPi)

The dry weight (DW) of roots and aerial parts (including shoots and leaves) were determined with standard methods. The fresh root and leaf samples at different time points after +Pi and -Pi treatments were weighted separately, rinsed in distilled water and dried, frozen, and ground in liquid nitrogen. Approximately 200 mg of grounded samples were suspended in 2 ml distilled water overnight, and centrifuged to pellet the cellular debris. Subsequently the supernatant was assayed for SPi using phosphomolybdate colorimetric assay [64].

### Quantitative real-time PCR

Total RNA were extracted from roots and leaves with a miRcute^TM^ miRNA Isolation Kit (Tiangen, http://www.tiangen.com) according to the manufacturer’s protocol. RNA samples were treated with RNase-free DNase I (Invitrogen, http://www.invitrogen.com) to avoid amplification from genomic DNA. The first cDNA strand was synthesized from total RNA using the PrimerScript RT Enzyme (TaKaRa, http://www.takara-bio.com/). The housekeeping gene *Glyma17g239000* (*GmEF1a*) was used as an endogenous control to normalize the samples [65]. Quantitative real-time PCR (qRT-PCR) was performed using SYBR Premix EX Taq^TM^ (TaKaRa). cDNA sequences and genomic sequences of *GmPLDZ2*, *GmIPS1*, *GmNRT2*, *GmHAK1*, and *GmSult1* were downloaded from Phytozome, and specific primer pairs were designed with PerlPrimer [66] as listed in Additional file 14. All reactions were run on a Rotor-Gene 3000 (Corbett Research, Australia). Reaction conditions for thermal cycling were based on standard methods.

### Construction of soybean small RNA libraries and degradome library and sequencing

The samples after harvested were immediately frozen in liquid nitrogen and stored at -80°C until RNA extraction. After RNA isolation, equal amounts of total RNA from roots and leaves at 6 h, 12 h, 7 d, and 14 d of Pi-replete or Pi-depleted treatments were mixed. Fragments of 18-30 bases were purified from 10 *μ*g total RNA mixture using a Novex 15% TBE-Urea gel. The 5’ and 3’ adaptors (Illumina) were added to the ends of fragments. Reverse transcription PCR (RT-PCR) was performed using a RT-PCR kit (Invitrogen). PCR products were purified and quantified for Illumina sequencing with a Solexa sequencer in Shenzhen Huada Gene Sci-Tech Company (Shenzhen, China).

The soybean Pi-depleted roots RNA degradome library was constructed as previously described [20,67,68]. The total RNA was extracted as described above. In brief, poly(A) RNA was extracted from 200 *μ*g of total RNA using miRcute^TM^ miRNA isolation Kit (Tiangen). A 5’ RNA adapter containing a MmeI recognition site was ligated to the poly(A) RNA possessing a 5’-phosphate with T4 RNA ligase (Ambion). The ligation products were purified and amplified by 20 PCR cycles and gel-purified for SBS sequencing in the Shenzhen Huada Gene Sci-Tech Company.

### Identification of miRNAs

Small RNA reads and degradome reads were both generated through Illumina Genome Analyzer II high- throughput sequencing. After sequencing, low quality reads and clip adapter sequences from raw data were removed via software developed by BGI ( http://www.genomics.cn). Small RNA libraries were then constructed from, small RNAs ranging from 18-31 nt, and then mapped to the soybean genome using SOAP2 [69]. Unique RNA sequences that perfectly matched the genome were subjected to subsequent analysis. The RNA reads showing sequences identical to known miRNAs from miRBase ( http://www.mirbase.org) were selected as the miRNA dataset for soybean. Further sequences matching non-coding rRNA, tRNA, snRNA and snoRNA in the Rfam database were removed. Then the reads overlapping with exons of protein coding genes were excluded to avoid mRNA contamination. The remaining sequences were considered to be candidate miRNA for further bioinformatics analyses.

Because miRNA precursors have a hairpin structure, 150-200 nt of the sequence flanking the genomic sequences of small RNAs was extracted from Phytozome ( http://www.phytozome.org). The MIREAP pipelinewas then used to analyze structural features to identify new miRNA candidates ( https://sourceforge.net/projects/mireap/). The resulting structures, with minimal matched nucleotide pairs of miRNA and miRNA* exceeding 16 nt and with maximal size differences of miRNA and miRNA* up to 4 nt, were retained as new miRNA candidates. The filtered pre-miRNA sequences were folded again using MFOLD and checked manually [70].

### Stem-loop quantitative real time PCR

Stem-loop specific reverse transcription was carried out according to the previously described Methods [25,71]. Reverse transcription reactions were performed using total RNA from soybean roots, and leaves grown under different nutrient conditions as indicated above. The gma-miR156b was used as a reference miRNA gene to normalize samples as previuously described [71]. Stem-loop specific reverse transcription was basically carried out according to the methods described [71]. The reactions contained 1 *μ*g of total RNA, and each reaction was primed with a pool of 0.25 *μ*M 5 gene-specific stem-loop primers. The RNA and primers were mixed with RNase-free water up to 10 *μ*l and incubated at 70°C for 5 min followed removed to ice-cooling immediately. Then, 6 *μ*l 5RT-Buffer, 1 *μ*l 5 mM dNTP, 0.5 *μ*l RNA Inhibitor, and 1 *μ*l 200 U MML-V RT Enzyme (Promega) were added and supplemented up to a final volume 30 *μ*l with RNase-free water. Synthesis was performed at 42°C for 30 min on a Veriti Thermal Cycler (Applied Biosystem), and inactivation of the enzyme was performed at 85°C for 5 min. Samples were then held at 4°C. All cDNA samples were 50-fold diluted with RNase-free water before being used as templates in RT-qPCR analysis. All primers used in stem-loop RT-PCR are listed in Additional file 14.

### RLM-5’ RACE

To determine the cleavage sites of miRNA on target genes, RLM-5’RACE was employed. Total RNA was extracted from Pi-depleted soybean leaves and roots with the miRcute miRNA Isolation Kit (TIANGEN, http://www.tiangen.com) as described by the manufacturer. Then, extracted total RNAs were ligated with 5’RACE oligo adaptors, and the reverse transcription was carried out based on the GeneRacer kit (Invitrogen, http://www.invitrogen.com). The first found PCR was carried out with 5’RACE general PCR and outer gene-specific PCR, and the next PCR was run using the diluted initial PCR reaction and inner 5’ RACE and gene-specific primers (GSP). PCR products were cloned and sequenced according to standard methods. Additional file 14 lists 5’ RACE and gene-specific PCR primers.

### Identification of target genes for miRNAs

After excluding low quality reads, reads with 5’ primer contaminants, reads without 3’ primer, reads without the insert tag, and reads shorter than 18 nt were removed. The remaining 20-21 nt-long reads with high quality were collected for subsequent analyses. Degradome tags were analyzed for expression and distribution on the soybean genome using SOAP2 [69]. Raw sequences were first normalized to reads per 10 million (RP10M), and identical degradome sequences with single base over 0.7 percentage in the clean reads was classified as polyN. Then, distinct reads that perfectly matched soybean cDNA sequences were further analyzed. The Pairfinder.pl script developed by the BGI Degradome group was used to align clean sequences to soybean known miRNAs from miRBase and miRNAs identified in this study. All alignments with scores up to 7 and no mismatches at the cleavage site (between the 10th and 11th nucleotide) were considered candidate targets.

### Analysis of miRNA promoter and *cis*-acting elements

Based on the pre-miRNA sequences of soybean miRNAs identified and perfectly mapped to the soybean genome ( http://www/phytozome.net/soybean) in this study, and not including pre-miRNA sequences curated in miRBase but not found in the present study, 2 kb sequences upstream of the pre-miRNAs were downloaded from Phytozome using the soybean genome browser. These sequences were used to predict transcription start sites (TSSs) and TATA-boxes, and analyze P-responsive *cis*-elements [31].

### Statistical analysis of data

All data for ratio of root to shoot, soluble phosphorus, and relative expression level of genes and mature miRNA are from experiments with three biological replicates. All data were analyzed using Origin 7.5 (OriginLab Corporation, USA) for calculating means and SEs, and SAS 6.2 (SAS Institute, USA) for ANOVA analyses.

## Abbreviations

A: Adenosine; AGO: Argonaute; C: Cytosine; CTK: Cytokinin; Cu: Copper; DCL: Dicer-like protein; DW: Dry weight; Fe: Iron; G: Guanosine; GO: Gene Ontology; GST: Glutathione S-transferase; K: potassium; miRNA: microRNA; N: Nitrgone; nov: novel; P: Phosphorus; POD: Peroxidase; PPR: Pentatricopeptide repeat; qRT-PCR: Quantitative real time PCR; RACE: rapid amplification of cDNA ends; RLM-5’ RACE: RNA ligase mediated 5’rapid amplification of cDNA ends; ROS: Reactive oxygen species; RT: Reverse transcription; S: sulfur; SBS: Sequencing-by-synthesis; siRNA: small interfering RNA; snRNA: small nuclear RNA; snoRNA: small nucleolar RNA; TF: Transcription factor; TPM: Transcripts per million.

## Competing interests

The authors declare that they have no competing interests.

## Authors’ contributions

JXW, FX, and HL designed the experiments. FX, QL, LYC, and JBK performed the experiments. JXW, FX and TW analyzed the data. JXW, TW, FX, and HL wrote the paper. All authors read and approved the final manuscript.

## Supplementary Material

Additional file 1 **Effects of phosphorus (P) starvation on dry weight ratio of roots to shoots (A), soluble inorganic phosphate (SPi) concentration (B), relative expression of *GmPLDZ2* (C) , and *GmIPS1* (D).** Asterisk above bar indicates the difference of phosphate-replete (+Pi) and phosphate-deplete (-Pi) is significant (^*^ : *P *<0.05; ^** ^: *P *<0.01; ^*** ^: *P *<0.005). Click here for file

Additional file 2 Categories of small RNAs origin in 4 small RNA libraries. Click here for file

Additional file 3 Percentage of 18-24 nt small RNAs in four small libraries from leaves and roots, respectively. Click here for file

Additional file 4 **The first nucleotide bias of small RNAs.** The first nucleotide bias of small RNAs ranged from 18 to 25 nt in four libraries: A:leaf+Pi (HPL); B: leaf-Pi (LPL); C:root+Pi (HPR); D:root-Pi (LPR).G, guanosine; C, cytosine; U, uridine; A, adenosine. Click here for file

Additional file 5 **Alignment of *GmIPS1 *with mature gma-miR399a-d and miR399e.** Three bulge formed in the central region. *GmIPS1 *CR, *GmIPS1* complementary region with miR399; *GmIPS1* (Gm10:5,886,477..5,887,249), *GmIPS2* (Gm13:24,562,834..24,562,257), *GmIPS3* (Gm03:41,903,760..41,903,360), *GmIPS4* (Gm19:44,409,842..44,410,371) were found in *Glycine max* genome v1.0 (http://www.phytozome.org), Accession number for their transcripts in TIGR are TA44356_3847, TA73486_3847, BF596594, TA56598_3847, respectively. Click here for file

Additional file 6 Statistics of degradome library from -Pi root RNA samples. Click here for file

Additional file 7 Target genes of conserved soybean miRNAs. Click here for file

Additional file 8 Target genes of less-conserved soybean miRNAs. Click here for file

Additional file 9 Targer genes of novel soybean miRNAs. Click here for file

Additional file 10 **GO analysis of the target genes for 126 soybean miRNAs.** The GO analysis of the 154 target genes base on their involved biological process for all 126 soybean miRNAs in this study was performed with AgriGO [29] according to the default settings (http://bioinfo.cau.edu.cn/agriGO/analysis.php). Click here for file

Additional file 11 Putative transcription start sites (TSSs) and TATA-boxes in the upstream region of soybean miRNA genes. Click here for file

Additional file 12 **Distribution of TSSs and TATA-boxes in different promoter region of soybean pre-miRNAs.** The 2-KB upstream region of 126 pre-miRNAs were download from Phytozome, the distribution of TSSs and TATA-boxes were counted in different region of the 2-kb long upstream region. Click here for file

Additional file 13 **List of *cis*-elements in the upstream region of soybean miRNA genes.** Click here for file

Additional file 14 List of primers used in qRT-PCR and stem-loop qRT-PCR. Click here for file
